# A factorial multi‐layer analysis reveals non‐additive sulfur–iron interactions shaping metabolic and transcriptional responses in durum wheat

**DOI:** 10.1111/nph.71507

**Published:** 2026-08-13

**Authors:** Eleonora Coppa, Mutsumi Watanabe, Moez Maghrebi, Giulia Quagliata, Alessandro Bruschini, Rainer Hoefgen, Youry Pii, Stefano Cesco, Gianpiero Vigani, Stefania Astolfi

**Affiliations:** ^1^ Department of Agriculture and Forest Sciences (DAFNE) University of Tuscia 01100 Viterbo Italy; ^2^ Department of Biological Sciences Nara Institute of Science and Technology Nara 630‐1104 Japan; ^3^ Max Planck Institute of Molecular Plant Physiology Potsdam 14476 Germany; ^4^ Department of Life Sciences and Systems Biology University of Torino 10135 Torino Italy; ^5^ Faculty of Agricultural, Environmental and Food Sciences Free University of Bolzano Bolzano 39100 Italy

**Keywords:** amino acid metabolism, asparagine, citrate, durum wheat, factorial multi‐omics, iron–sulfur crosstalk

## Abstract

- Reciprocal iron–sulfur regulation in durum wheat converges on a few metabolic hubs that integrate nutrient status with plant growth and performance.
- Iron and sulfur availabilities were factorially modulated in hydroponically grown wheat to establish how each nutrient and their interaction shape growth, ionome, amino acid and organic acid metabolism, and the expression of key S‐ and Fe‐homeostasis genes.
- Sulfur availability strongly shaped plant responses to iron availability, determining biomass allocation, ionomic patterns, and the expression of sulfate transporters, sulfur‐assimilatory enzymes, and iron homeostasis genes. Metabolomics highlighted citrate, O‐acetylserine, and methionine as root metabolites most consistently associated with combined Fe × S treatments. Correlation analysis linked these variables with markers of sulfate transport and assimilation, defining a reproducible interaction signature across biological layers. Sulfur deficiency, especially when combined with low iron, also increased free asparagine accumulation in vegetative tissues, indicating a strong reallocation in nitrogen metabolism under dual stress.
- Sulfur availability is a major modulator of iron‐responsive phenotypic, transcriptional, and metabolic traits in durum wheat and provides a systems‐level framework for future functional studies of Fe/S crosstalk.

## Graphical abstract

Working model of S‐dependent Fe stress integration in durum wheat. S availability acts as the main factor defining the metabolic and transcriptional state of the plant, whereas Fe mainly affects the intensity of these responses. This is particularly evident for OAS, methionine, citrate and asparagine, which accumulated most strongly under combined S and Fe deficiency and progressively decreased as Fe availability increased. The coordinated behavior of these compounds, together with their association with S assimilation and Fe‐homeostasis genes, highlights a coordinated root‐centered network that integrates S and Fe nutrition.

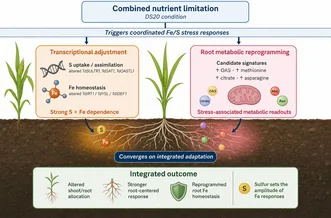

## Introduction

Nutrient availability is a major determinant of plant growth, metabolism, and overall fitness. Among the essential mineral elements, sulfur (S) and iron (Fe) are critical for a wide range of physiological processes. Sulfur is a key component of amino acids like cysteine and methionine, which are fundamental for protein synthesis, and it is also involved in the synthesis of coenzymes and secondary metabolites (Kopriva *et al*., 2009). Iron is crucial for photosynthesis, respiration, and nitrogen (N) fixation, acting as a cofactor for numerous enzymes and proteins in redox processes (Briat *et al*., 2007). Deficiency of either S or Fe can severely impair plant health and vigor, reduce yields, and affect the nutritional quality of crops (Schmidt *et al*., 2020; Astolfi *et al*., 2021; Narayan *et al*., 2023).

Sulfur deficiency has become increasingly common due to decreased atmospheric deposition and the use of low‐S fertilizers (Zhao *et al*., 1999), while Fe availability is frequently limited in calcareous and alkaline soils, which cover more than 30% of the Earth's surface, because of its low solubility (Marschner, 2012; FAO, 2015). Furthermore, due to their shared roles in metabolic pathways and redox reactions, the availability and metabolism of S and Fe are intricately linked, and a deficiency in one of the two nutrients induces physiological modifications that allow the plant to adjust the uptake and utilization of the other (Astolfi *et al*., 2021). This interplay between the two nutrients is particularly relevant in cereals like durum wheat (*Triticum durum*), a global staple crop.

Previous studies have indicated that Fe uptake and accumulation in plants strongly depend on S availability in the growth medium (Zuchi *et al*., 2012), while adaptation to Fe deficiency requires adjustments in S uptake and assimilation rates (Ciaffi *et al*., 2013). Furthermore, modulating S availability can significantly influence plant responses to Fe deficiency. In durum wheat, a supra‐optimal S supply was shown to promote Fe accumulation in the shoots and enhance release of phytosiderophores (PS), hence improving root Fe use efficiency (Zuchi *et al*., 2012; Hawkesford *et al*., 2014; Celletti *et al*., 2016). More recently, certain durum wheat genotypes were found to enhance Fe accumulation in grains under combined supra‐optimal S availability and Fe deficiency, or under a high S supply alone (Bruschini *et al*., 2025), highlighting the agronomic relevance of Fe/S interaction for crop nutritional quality.

This relationship has been largely attributed to the role of S in the biosynthesis of PS, since methionine is a common precursor for both PS and nicotianamine (Mori & Nishizawa, 1987). Indeed, in barley, S deficiency reduced PS release (Astolfi *et al*., 2006) and modulated the expression of HvYS1, the specific transporter of Fe(III)‐PS complexes (Astolfi *et al*., 2010), while restoration of S supply enables full recovery of Fe deficiency responses (Astolfi *et al*., 2010, 2012). Furthermore, a transcriptomic analysis of durum wheat roots has shown that S deficiency downregulates genes encoding formate dehydrogenase, leading to impaired formate removal and reduced NADH availability for PS biosynthesis (Zamboni *et al*., 2017), ultimately constraining Fe acquisition (Mori, 1999).

Beyond PS biosynthesis, Fe and S interactions are also mediated through Fe–S cluster biogenesis, a complex process involving multiple cellular compartments, including chloroplasts, mitochondria, and the cytosol (Balk & Schaedler, 2014). Mitochondria have been proposed as central hubs for coordinating Fe and S homeostasis in response to nutritional stress (Vigani & Briat, 2016).

This complex interaction is further mediated by specific metabolic adjustments, such as the production of organic acids (Balk & Pilon, 2011; Vigani & Briat, 2016). Citrate, a tricarboxylic acid (TCA) cycle intermediate, plays a well‐established role in Fe acquisition and transport, particularly in tomato (Coppa *et al*., 2018; Vigani *et al*., 2018). Moreover, increased citrate levels have been associated with modulation of TOR (Target of Rapamycin) activity in both shoots and roots, suggesting a regulatory role for citrate in plant adaptation to Fe and S deficiencies (Coppa *et al*., 2023). However, the contribution of citrate to the coordination of S and Fe metabolic responses, especially under combined nutrient stress, remains poorly understood.

Understanding how these nutrients interact at the metabolic level is crucial for developing strategies to improve crop yield and nutritional quality, especially in the context of declining soil fertility and the additional pressures imposed by climate change.

To dissect the mechanisms underlying Fe/S crosstalk, this study was designed to systematically explore plant responses across a matrix of Fe and S availabilities. By exposing durum wheat plants to multiple Fe concentrations under contrasting S regimes, ranging from deprivation to supra‐optimal supply, we aimed to determine how the magnitude and direction of metabolic responses are reciprocally modulated by the nutritional status of the other element. By integrating phenotypic, ionomic, metabolomic and transcriptional datasets across a factorial nutrient design, this study provides a comprehensive system‐level view of Fe/S interaction in a cereal crop. This factorial approach allows discrimination between additive responses to individual nutrient limitations and non‐additive, interaction‐driven responses that reveal true metabolic integration. Through this integrated approach, we aim to identify key metabolic features and coordinated responses associated with Fe/S interaction and to highlight their agronomic and possible food safety implications.

## Materials and Methods

### Plant growth conditions and sampling

Durum wheat (*Triticum durum* L. cv Svevo) seeds were germinated on moistened paper in the dark at 20°C for 3 d. The seedlings were then transferred to plastic pots (12 seedlings per pot) filled with 2.2 l of continuously aerated nutrient solution (NS) (Zuchi *et al*., 2012). The experiment involved three different sulfate (S) supplies (0, 1.2, and 2.4 mM, representing deficient, adequate, and supra‐optimal levels, namely DS, CS and ES, respectively) and three Fe(III)‐EDTA levels (20, 50, and 80 μM). The S‐deficient NS was prepared by replacing sulfate salts with appropriate amounts of chloride salts. The S application rates were based on previous studies, with 1.2 mM chosen as the adequate supply (consistent with other grasses) and 2.4 mM as the supra‐optimal supply (double the adequate concentration) (Astolfi *et al*., 2004, 2006; Bruschini *et al*., 2025). Plants were grown in a climate chamber under 200 μmol m^−2^ s^−1^ PAR at the leaf level, with a 14 h : 10 h, light : dark regime (temperature 27°C : 20°C, day : night; relative humidity 80%). Wheat shoots and roots were sampled 14 d after sowing.

### Leaf greenness

Leaf greenness was estimated non‐destructively by a portable Chl meter (SPAD Meter—502 Plus, Konica Minolta Co., Osaka, Japan). Measurements were taken on the youngest fully expanded leaf of three plants (three leaves per plant) and the results were expressed as SPAD units.

### Root morphological analysis

At harvest, the root system architecture was evaluated using Winrhizo software from images taken by placing roots in a water‐filled plexiglass tray to minimize overlap. The parameters measured included root length, surface area, volume, average diameter, and number of root tips.

### Determination of macro‐ and micronutrient concentrations

Oven‐dried (80°C for 48 h) root and shoot tissues were used to determine the concentrations of macronutrients (Mg, Ca, K) and micronutrients (Fe, Mn, Cu, Zn, Mo). Samples were mineralized using a microwave digestion system (Multiwave Go Plus, Anton Paar GmbH, Graz, Austria) with a mixture of ultrapure HNO_3_ (69.5%) and HCl (37%). After mineralization and filtration, the samples were diluted and their elemental concentrations were measured by inductively coupled plasma‐mass spectroscopy (ICP‐MS; Agilent ICP‐MS 7850, Santa Clara, CA, USA).

### Metabolomic analysis

Root and shoot tissues were ground in liquid nitrogen, and 100–150 mg of fresh material was used for metabolite extraction and analysis following Lisec *et al*. (2006). Samples were homogenized in a ball mill, and metabolites were extracted with methanol containing ribitol as an internal standard. After heating and centrifugation, the polar phase was collected, mixed with chloroform and water, and centrifuged again. The aqueous phase was dried under vacuum and derivatized with methoxyamine hydrochloride in pyridine, followed by N‐methyl‐N‐(trimethylsilyl)trifluoroacetamide. Derivatized samples were analyzed by GC‐TOF/MS using fatty acid methyl esters as retention time standards. Data processing and metabolite quantification were performed using the tagfinder software.

Cysteine analysis was performed as described in Zuchi *et al*. (2015). Briefly, 50 mg of homogenized shoot and root tissue was extracted in 100 μl of 0.1 M HCl and 1% (w/v) polyvinylpolypyrrolidone (PVPP). After placing on ice, the extract was derivatized by adding 150 μl of 0.25 M 2‐(Cyclohexylamino)ethanesulfonic acid (CHES) buffer (pH 9.4) and 20 μl of 25 mM monobromobimane (MBB). The reaction proceeded for 15 min at 4°C in the dark and was terminated with 100 μl of 100 mM methanesulfonic acid. Following centrifugation (14 000 ***g*** for 15 min at 4°C), the supernatant was diluted 1 : 3 in running buffer (0.25% acetic acid, pH 4.0; 8% methanol) and analyzed via high‐performance liquid chromatography. Separation was performed using an increasing methanol gradient at pH 4.0 with fluorescence detection (*λ*ex 380 nm/*λ*em 480 nm) according to Hubberten *et al*. (2012). Cysteine concentrations were quantified using external standards processed alongside the samples.

### Total RNA extraction and gene expression analysis by using qRT‐PCR

Total RNA was isolated from roots and shoots using TRIzol® Reagent (Life Technologies, Carlsbad, CA, USA) and subsequently purified with the PureLink® RNA Mini Kit (Life Technologies), following the manufacturer's protocol. Genomic DNA contamination was eliminated on‐column using PureLink® DNase (Life Technologies). First‐strand cDNA synthesis was performed with the SuperScript™ III First‐Strand Synthesis SuperMix for qRT‐PCR (Invitrogen), according to the supplier's instructions.

Quantitative real‐time PCR (qRT‐PCR) analysis of genes related to S and Fe homeostasis (*TdSULTR1.1*, *TdSULTR1.3*, *TdSAT1*, *TdOASTL1*, *TdIDEF1*, *TdIRT1* and *TdYSL1*) was conducted using the synthesized cDNA as template in a 20 μl reaction mixture containing GoTaq® qPCR Master Mix (Promega) and gene‐specific primers (listed in Supporting Information Table S1). Ensembl transcript identifiers and genomic locations of the selected sequences are provided in Table S2. Amplification was carried out on a QuantStudio 3 Real‐Time PCR System (Applied Biosystems). The relative expression of each gene was calculated using the 2^−ΔΔCt^ method (Livak & Schmittgen, 2001), with *TdACTIN* serving as the internal reference gene.

### Statistical analysis

Each reported value represents the mean ± SD (SD) of three independent biological replicates (*n* = 3), with the exception of metabolomics data, which utilized six biological replicates (*n* = 6). For all treatments, individual pots served as the biological unit. Data were analyzed using two‐way ANOVA to evaluate the effects of two independent factors on the dependent variables. To identify significant differences in dependent variables induced by the growth conditions, one‐way ANOVA was followed by Tukey's *post hoc* tests (*P* < 0.05). The analysis and the plots were carried out using R software (Rstudio, V. 2023.12.0 + 369) and the packages: ggplot2 (v.3.5.1), dplyr (v.1.1.4), multcompview (v.0.1‐10) and ggpattern (v.1.1.4). Hierarchical clustering on root traits and ionomic datasets was performed with the full method and Euclidean distance measurement in R (V. 2023.12.0 + 369), using the packages pheatmap (v.1.0.12), rcolorbrewer (v.1.1‐3) and gplots (v.3.1.3.1).

The correlation networks were performed using R software (Rstudio, V. 2023.12.0 + 369) and the packages: tidyverse (v.2.0.0), corrr (v.0.4.4), igraph (v.1.5.1), ggraph (v.2.1.0) and ggrepel (v.0.9.5). Correlation networks were constructed by discriminating variables with an *R* ^2^ value lower than 0.3.

To summarize the relative contribution of S, Fe and S × Fe across traits, we reported partial Eta squared (η_p_^2^) from two‐way ANOVA, a widely used effect‐size metric for factorial designs (Fernández‐Marín *et al*., 2020; Barmeier *et al*., 2021). Statistical analyses were performed in R using RStudio (v. 2023.12.0 + 369). Two‐way ANOVA models were fitted using the base R stats package, η_p_^2^ values were calculated using effect size (v.1.0.2), and heatmaps were generated using ggplot2 (v.3.5.1).

## Results

### Sulfur status modulates growth responses and nutrient partitioning in response to Fe availability

**Fig. 1:**
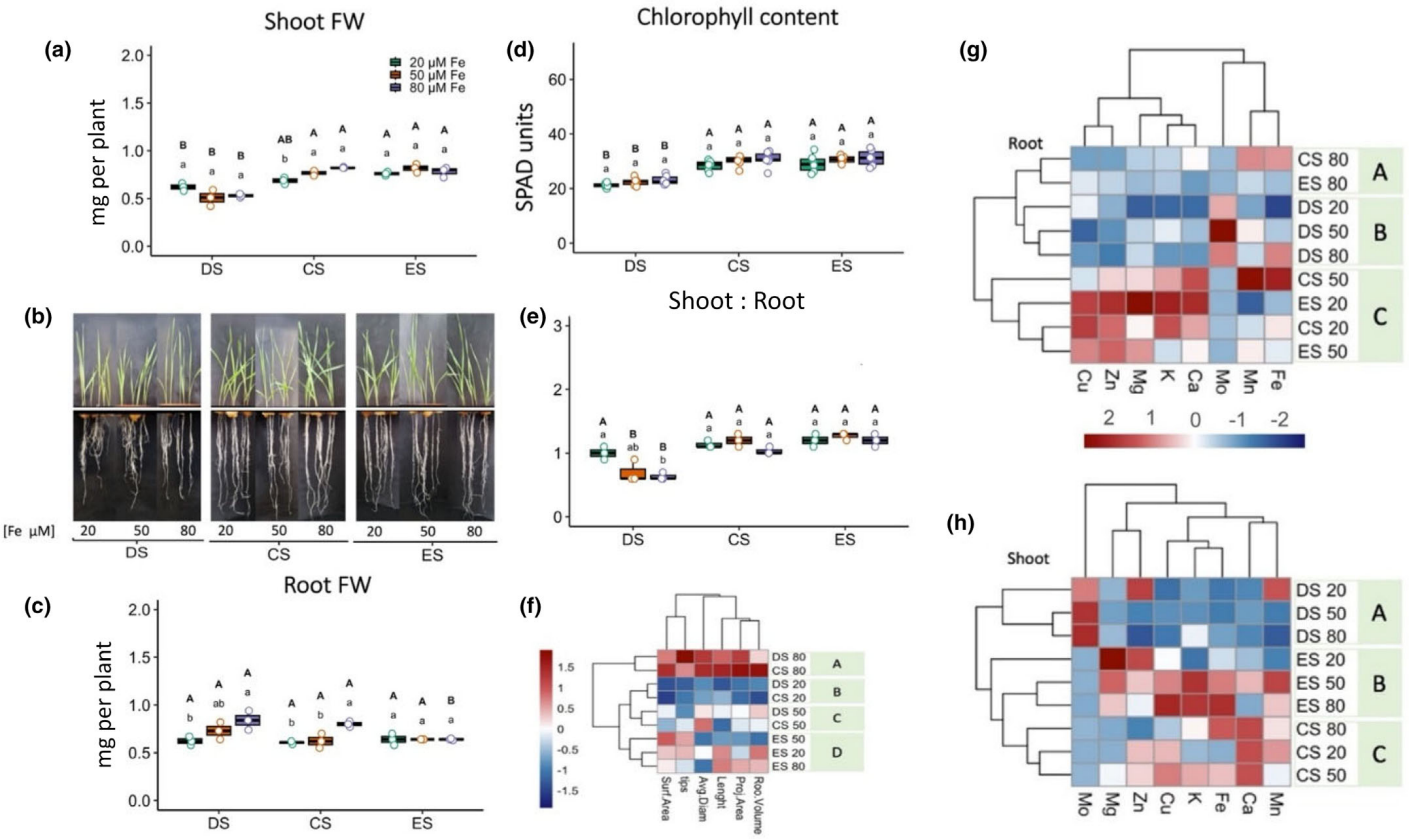
Phenotypic response of durum wheat to varying S and Fe supply. Plants were grown hydroponically for 2 wk at 0 (DS: deficient S supply), 1.2 (CS: control S supply), and 2.4 mM (ES: supra‐optimal S supply) sulfate, combined with 20, 50, and 80 μM Fe(III)‐EDTA. Shoot (a) and root (c) fresh weight; representative images of 2‐wk‐old seedlings (b); Chl content (d); shoot‐to‐root ratio (e). Boxplots show the median, interquartile range, and data dispersion, while points represent biological replicates (*n* = 3 for a–f; *n* = 6 for d). Data were analyzed using one‐way ANOVA followed by Tukey's *post hoc* test (*P* < 0.05). Different capital letters indicate significant differences among sulfate levels (S effect) within the same Fe concentration, while lowercase letters indicate significant differences among Fe(III)‐EDTA concentrations (Fe effect) within the same sulfate level (Tukey's HSD test, *P* < 0.05). Hierarchical clustering heatmap of root traits dataset (f) and root (g) and shoot (h) ionomic profiles. The color scale from blue (low) to red (high) represents changes across treatments; distinct clusters are labeled A–D.

Fig. 1 shows plant biomass allocation (a, b, c and e) and root morphological traits (f) in response to increasing Fe supply (20, 50, 80 μM) under three S regimes: S‐deficient (DS), optimal S (CS), and supra‐optimal S (ES). Under S deficiency (DS), root fresh weight showed a pronounced positive response to increasing Fe availability, nearly doubling (from 0.50 to 0.89 g per plant) between 20 and 80 μM Fe (Fig. 1c). This trend diminished under CS condition and disappeared entirely under ES condition, where root biomass remained relatively stable across Fe treatments (Fig. 1c). Shoot fresh weight (Fig. 1a) was primarily affected by S supply, increasing from DS to CS treatment, regardless of Fe concentration, while no further increase was observed under ES. Interestingly, under optimal S conditions (CS), shoot biomass increased progressively with Fe supply, indicating that adequate S availability is required to fully exploit Fe‐dependent growth responses (Fig. 1a). Consistently, the shoot‐to‐root ratio decreased sharply with increasing Fe under DS, reflecting preferential allocation to root growth, whereas it remained stable across Fe levels under CS and ES (Fig. 1e).

Root system architecture was analyzed, considering root length, volume, diameter, surface area, and number of tips (Fig. S1). Hierarchical clustering of root morphological traits separated the nine treatments into four distinct groups (Fig. 1f). All supra‐optimal S treatments (ES20, ES50 and ES80) clustered together, suggesting that high S supply largely overrides Fe effects on root architecture. On the other hand, under DS and CS conditions, Fe availability shaped root morphology. In particular, the 80 μM Fe treatments formed a cluster characterized by uniformly elevated trait values indicating a vigorous root development, while the 20 μM Fe treatments grouped separately with consistently reduced values, indicating inhibited root growth. The 50 μM Fe treatments occupied an intermediate cluster, exhibiting transitional morphological characteristics (Fig. 1f). Regarding the Chl content, Fig. 1(d) reveals no significant effect of Fe availability, but demonstrates a significant increase when S was supplied at supra‐optimal concentrations.

To determine whether these growth and architectural responses were associated with changes in nutrient accumulation, shoot and root ionomic profiles were analyzed (Figs 1g,h, S2).

Analysis of shoot ionomic profiles revealed a distinct S‐driven clustering pattern (Fig. 1g). All S‐deficient treatments (DS20, DS50, DS80) clustered together (Cluster A) and were characterized by high Mo accumulation, with additional enrichment of Zn and Mn observed under DS20 condition (Fig. 1g). Control S treatments (CS20, CS50, CS80) formed a distinct cluster (C) displaying balanced mineral profiles, featuring a pronounced Ca accumulation and low Mo levels (Fig. 1g). Supra‐optimal S treatments (ES20, ES50, ES80) grouped into an intermediate cluster (Cluster B) characterized by high Mg and reduced Mo levels (Fig. 1g). Within this cluster, ES50 and ES80 also exhibited increased levels of Cu, K, Fe, and Mn (Fig. 1g).

In roots, hierarchical clustering identified three distinct groups reflecting combined effects of S and Fe supply (Fig. 1h). Cluster A comprised CS80 and ES80 treatments and was characterized by generally low concentrations of most nutrients, with the exception of Fe and Mn, which were relatively elevated under CS80 (Fig. 1h). Cluster B encompassed all S‐deficient conditions (DS20, DS50, DS80), marked by an overall depletion of macro‐ and micronutrients, albeit an increased Mo concentration across all treatments and a pronounced rise in Fe accumulation at DS80 were observed (Fig. 1h). Finally, Cluster C grouped S‐sufficient treatments supplied with low to intermediate Fe (CS20, CS50, ES20, and ES50), distinguished by high Ca, K, Mg, Zn, and Cu levels, with elevated Fe and Mn specifically under CS50 (Fig. 1h).

### Sulfur status defines Fe‐dependent transcriptional regulation of S and Fe homeostasis

**Fig. 2:**
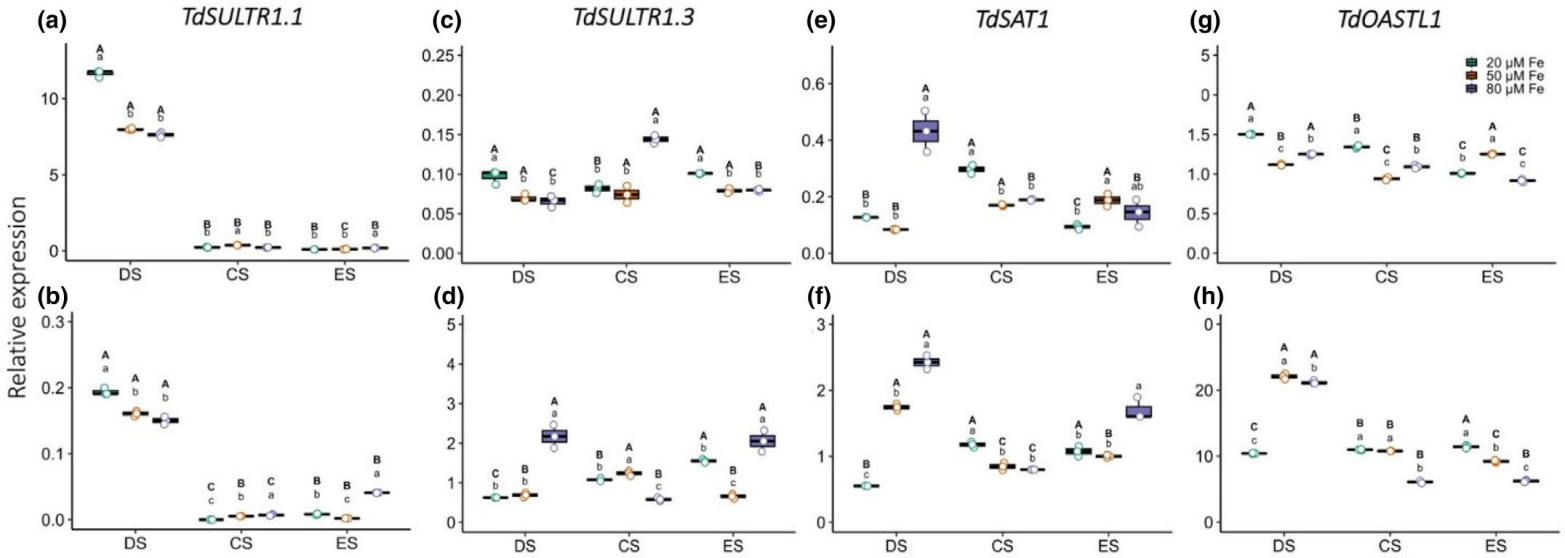
Expression profiles of S‐homeostasis genes in durum wheat. Plants were grown hydroponically for 2 wk at 0 (DS: deficient S supply), 1.2 (CS: control S supply), and 2.4 mM (ES: supra‐optimal S supply) sulfate, combined with 20, 50, and 80 μM Fe(III)‐EDTA. Root and shoot relative mRNA levels of TdSULTR1.1 (a, b), TdSULTR1.3 (c, d), TdSAT1 (e, f), and TdOASTL1 (g, h). Boxplots show the median, interquartile range, and data dispersion, while points represent biological replicates (*n* = 3). Data were analyzed using one‐way ANOVA followed by Tukey's *post hoc* test (*P* < 0.05). Different capital letters indicate significant differences among sulfate levels (S effect) within the same Fe concentration, while lowercase letters indicate significant differences among Fe(III)‐EDTA concentrations (Fe effect) within the same sulfate level (Tukey's HSD test, *P* < 0.05).

**Fig. 3:**
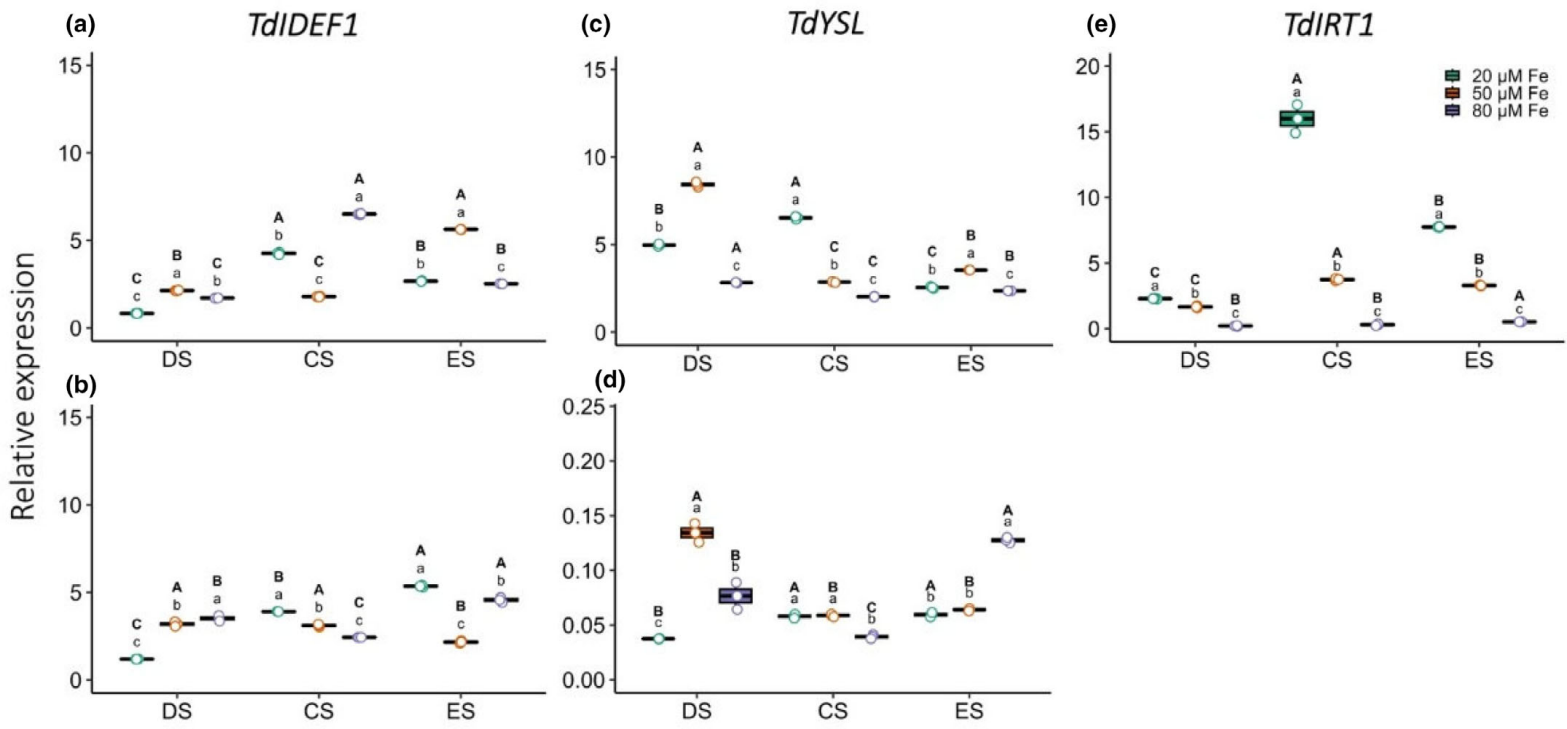
Transcriptional response of Fe‐homeostasis genes in durum wheat. Plants were grown hydroponically for 2 wk at 0 (DS: deficient S supply), 1.2 (CS: control S supply), and 2.4 mM (ES: supra‐optimal S supply) sulfate, combined with 20, 50, and 80 μM Fe(III)‐EDTA. Root and shoot relative expression levels of TdIDEF1 (a, b), and TdYSL1 (c, d) and TdIRT1 (e). The expression of TdIRT1 was not detectable in shoot tissues across all treatments. Boxplots show the median, interquartile range, and data dispersion, while points represent biological replicates (*n* = 3). Data were analyzed using one‐way ANOVA followed by Tukey's *post hoc* test (*P* < 0.05). Different capital letters indicate significant differences among sulfate levels (S effect) within the same Fe concentration, while lowercase letters indicate significant differences among Fe(III)‐EDTA concentrations (Fe effect) within the same sulfate level (Tukey's HSD test, *P* < 0.05).

Given the strong S‐dependent effects on growth, root architecture and nutrient partitioning, we next examined whether these responses were underpinned by coordinated transcriptional regulation of key genes involved in S and Fe homeostasis (Figs 2, 3). In particular, in the case of S metabolism, the sulfate transporters *TdSULTR1;1* and *TdSULTR1;3*, together with the genes encoding for the assimilatory enzymes *TdSAT1* (serine acetyltransferase) and *TdOASTL1* (*O*‐acetylserine(thiol)lyase) were considered. For Fe homeostasis, the transcription factor *TdIDEF1* (Fe deficiency‐responsive element‐binding factor 1), *TdIRT1* (Fe‐regulated transporter 1), and the yellow stripe‐like transporter *TdYSL* were targeted.

In roots, the high‐affinity sulfate transporter *TdSULTR1;1* was strongly induced by S deficiency, with the highest expression under DS at low Fe availability (20 μM Fe) and a progressive decline as Fe supply increased (Fig. 2a). Under CS and ES, *TdSULTR1;1* expression in roots remained very low across all Fe treatments. In shoots, a similar S‐dependent pattern was observed, although transcript abundance was *c*. 60 times lower than in roots (Fig. 2a). Under CS and ES, shoot expression was very low, with only a slight increase induced by 80 μM Fe under ES (Fig. 3b).

By contrast, root *TdSULTR1;3* also declined with increasing Fe under DS, whereas under CS it was significantly induced at 80 μM Fe; under ES, transcript levels remained relatively stable across Fe supplies (Fig. 2c). In shoots, TdSULTR1;3 was significantly upregulated at 80 μM Fe under both DS and ES treatments, while remaining comparatively low and unchanged under CS (Fig. 2d).

Expression of the assimilatory enzyme *TdSAT1* showed a strong interaction between S and Fe availability. In roots, S deficiency (DS) upregulated *TdSAT1* expression only at 80 μM Fe (Fig. 2e), whereas under CS conditions transcript levels peaked at low Fe (20 μM Fe) and declined with increasing Fe availability; ES resulted in intermediate expression levels (Fig. 2e). In shoots, *TdSAT1* displayed a pronounced Fe dose–response under DS, increasing from 0.55 at 20 μM Fe to 2.45 at 80 μM Fe (Fig. 2f). Conversely, under CS, shoot expression decreased with rising Fe supply, whereas ES conditions triggered significant upregulation specifically at 80 μM Fe (Fig. 2f).

For *TdOASTL1*, root expression showed the same pattern under DS and CS conditions, reaching the highest level at 20 μM Fe, followed by a modest decline at highest Fe concentrations (Fig. 2g). By contrast, under ES treatment, the expression of *TdOASTL1* maintained a stable levels across Fe treatments (Fig. 2g). Similarly to *TdSAT1*, shoot *TdOASTL1* expression was strongly upregulated by Fe under DS, peaking at 22 with 50 μM Fe and 21 with 80 μM Fe (Fig. 2). Under both CS and ES conditions, shoot expression levels decreased with increasing Fe supply, with the lowest expression detected at ES80 (Fig. 2h).

The expression patterns of both *TdIRT1* and *TdYSL* and the transcription factor *TdIDEF1* highlight their respective roles in Fe mobilization and Fe deficiency response regulation (Fig. 3).

The expression pattern of *TdIRT1* in roots showed its classic Fe deficiency response, with highest expression under CS at 20 μM Fe, declining sharply as Fe supply levels were increased (Fig. 3e). Under DS and ES, root *TdIRT1* remained much lower than under CS condition, although it was still consistently upregulated by Fe deficiency (Fig. 3e). Notably, the expression of *TdIRT1* was not detectable in shoots across all conditions, consistent with its role as a root‐specific Fe uptake transporter (Fig. 3).

In roots, DS triggered the highest expression of *TdYSL* at 50 μM Fe, with lower levels at both 20 and 80 μM Fe (Fig. 3c). Under CS, root *TdYSL* transcript abundance peaked at 20 μM Fe and declined with higher Fe, whereas under ES conditions its expression was low and stable across Fe treatments (Fig. 3c). In shoots, *TdYSL* expression was induced under DS at 50 μM Fe, as observed in roots, and strongly upregulated under ES at 80 μM Fe, whereas under CS no significant change was found across Fe supply (Fig. 3d).

In contrast to the root‐specific expression of *TdIRT1*, *TdIDEF1* showed significant expression in both roots and shoots, consistent with the expression profile of *TdYSL* (Fig. 3a,b). In roots, its transcription was upregulated by 50 μM Fe under both DS and ES conditions (Fig. 3a). Under control S (CS), *TdIDEF1* transcript levels reached the highest levels at the extremes of Fe availability (20 μM and 80 μM Fe), with a pronounced decline at 50 μM Fe (Fig. 3a). In shoots, *TdIDEF1* was most highly expressed under ES condition at the lowest Fe supply (20 μM), with lower levels under CS, especially at 80 μM Fe, and under ES, especially at 20 μM Fe (Fig. 3b).

Together, these transcriptional responses mirror the S‐dependent changes in biomass allocation and ionomic profiles, indicating S availability as a major determinant of the molecular regulation of both S assimilation and Fe homeostasis.

### Sulfur–iron interactions drive tissue‐specific metabolic signatures in roots and shoots

**Fig. 4:**
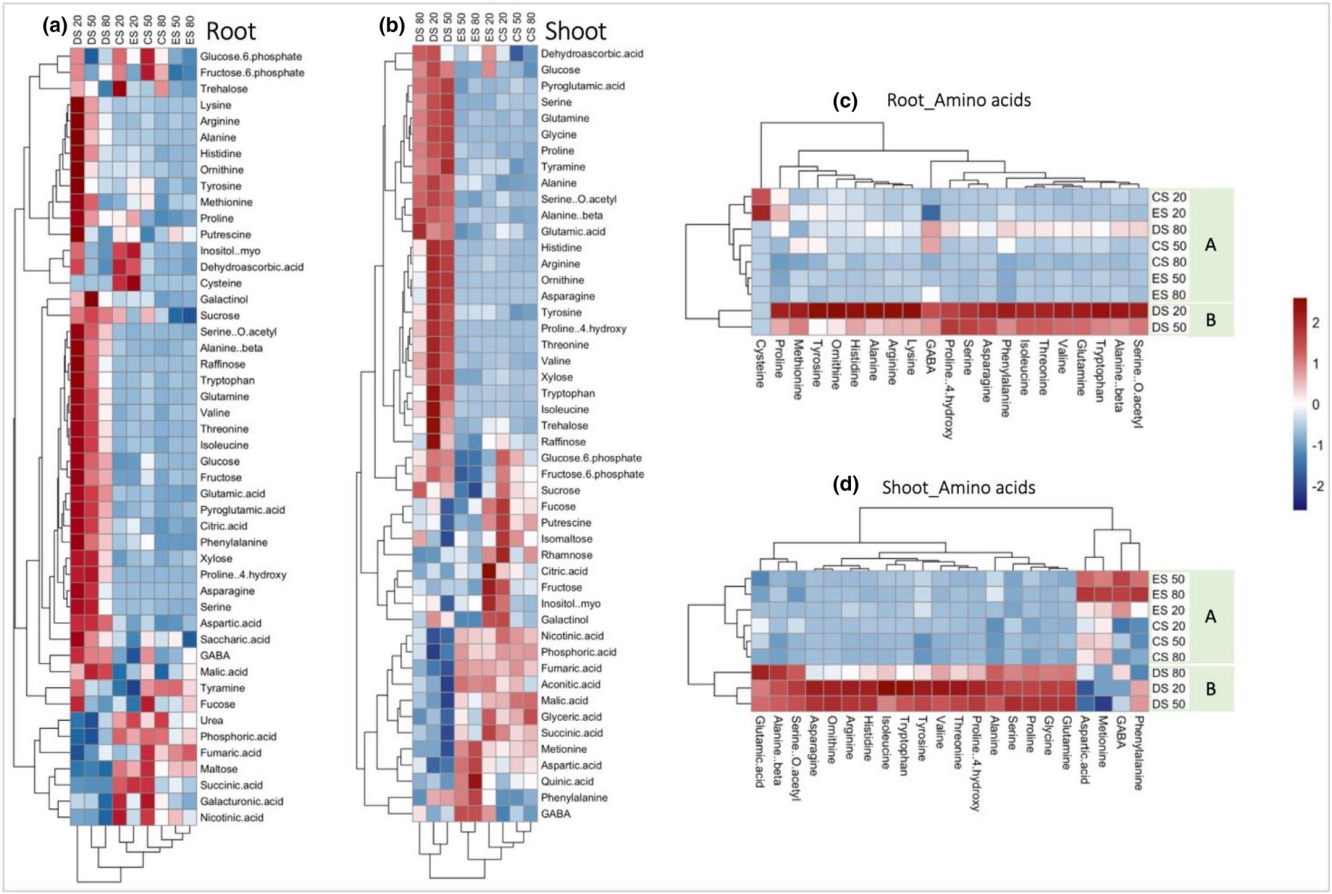
Metabolomic analysis of durum wheat under varying S and Fe supply. Plants were grown hydroponically for 2 wk at 0 (DS: deficient S supply), 1.2 (CS: control S supply), and 2.4 mM (ES: supra‐optimal S supply) sulfate, combined with 20, 50, and 80 μM Fe(III)‐EDTA. Hierarchical clustering heatmaps of root (a) and shoot (b) metabolite profiles. Detailed amino acid accumulation in roots (c) and shoots (d). The color gradient reflects relative concentration (*Z*‐score normalized peak intensity), ranging from blue (low) to red (high). Data are means of six biological replicates (*n* = 6). Capital letters A and B indicate the main clusters identified in the amino acid analysis.

Taken together, the S‐ and Fe‐dependent effects on growth, ionomic partitioning and gene expression indicate a complex interaction between these two nutrients. To determine how these coordinated responses are integrated at the metabolic level and to identify key metabolites acting as metabolic hubs, we next performed a comprehensive metabolomic analysis of roots and shoots using hierarchical clustering and heatmap visualization (Fig. 4). In both tissues, S availability emerged as the primary driver of metabolic reprogramming, with S‐deficient (DS) conditions being clearly separated from S‐sufficient treatments.

In roots, DS20 and DS50 conditions formed a distinct, tightly linked cluster, characterized by a marked accumulation of a large group of amino acids (e.g. serine, arginine, histidine, threonine, glycine, asparagine) and related compounds (e.g. putrescine, *O*‐acetylserine, inositol). Conversely, these two DS conditions show a strong depletion of several organic acids (e.g. citric acid, pyruvic acid, fumaric acid, succinic acid) and some sugars (e.g. fructose, glucose‐6‐phosphate). By contrast, control and excess S treatments (CS and ES) formed a distinct cluster displaying the opposite metabolic pattern, generally showing lower levels of the accumulated amino acids and higher levels of organic acids. Citric acid and malic acid displayed their lowest accumulation in DS 80, CS, and ES conditions (Fig. 4a).

In shoot, the metabolic profile showed a similar clustering pattern, albeit distinctive features could be highlighted (Fig. 4b). The amino acid group (e.g. glutamine, proline, tyramine, serine, O‐acetylserine) showed its highest relative accumulation in DS20, DS50, and DS80 conditions, suggesting a dominant influence of S deficiency. The DS cluster was also characterized by lower levels of several organic acids (e.g. citric acid, fumaric acid, succinic acid, malic acid) and some sugars (e.g. fructose‐6 phosphate, glucose‐6 phosphate, sucrose) (Fig. 4b). In contrast to the roots, the Fe(III)‐EDTA level of 80 μM under DS (DS80) was metabolically closer to the double‐deficiency conditions (DS20), especially in terms of *O*‐acetylserine and amino acid accumulation (Fig. 4b).

**Fig. 5:**
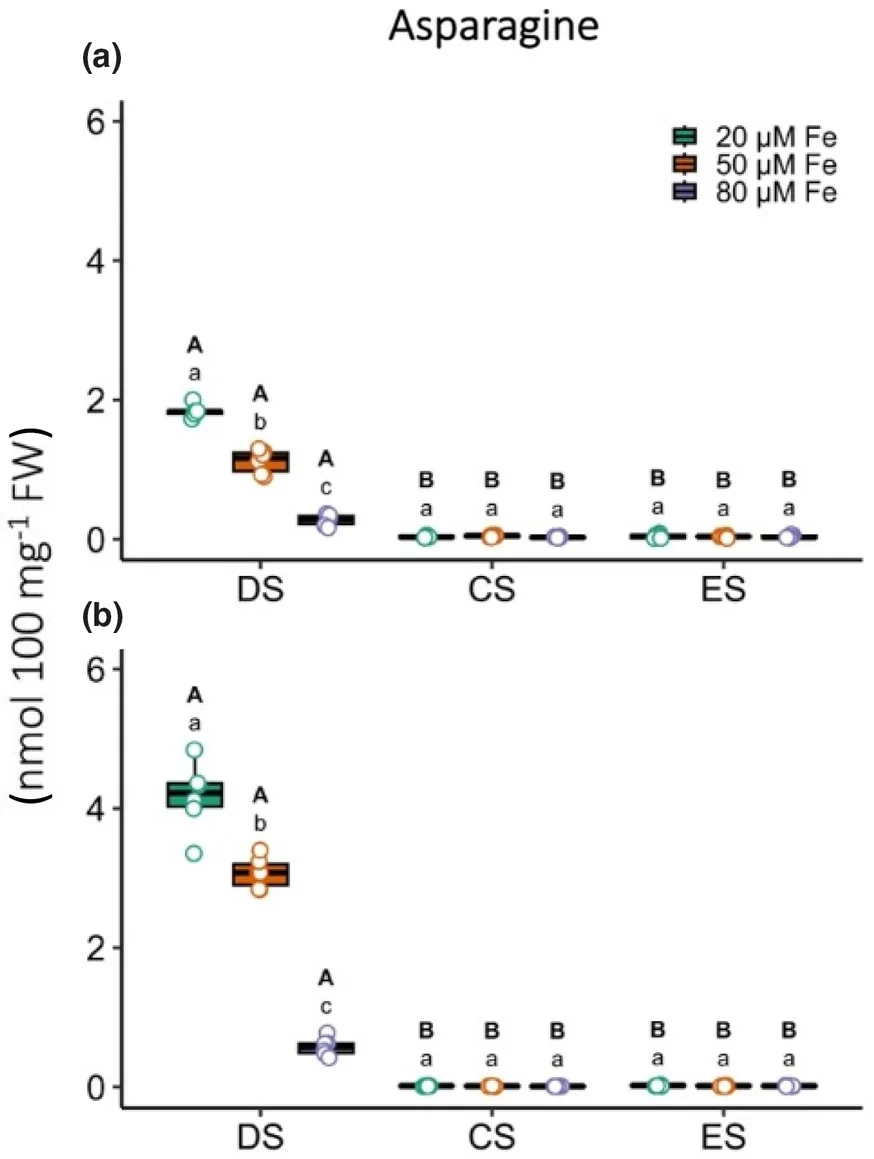
Asparagine concentrations in roots (a) and shoots (b) of durum wheat under varying S and Fe supply. Plants were grown hydroponically for 2 wk at 0 (DS: deficient S supply), 1.2 (CS: control S supply), and 2.4 mM (ES: supra‐optimal S supply) sulfate, combined with 20, 50, and 80 μM Fe(III)‐EDTA. Boxplots show the median, interquartile range, and data dispersion, while points represent biological replicates (*n* = 6). Data were analyzed using one‐way ANOVA followed by Tukey's *post hoc* test (*P* < 0.05). Different capital letters indicate significant differences among sulfate levels (S effect) within the same Fe concentration, while lowercase letters indicate significant differences among Fe(III)‐EDTA concentrations (Fe effect) within the same sulfate level (Tukey's HSD test, *P* < 0.05).

As highlighted by the hierarchical clustering (Fig. 4a,b), a clear distinction emerged in the metabolic response of amino acids in both root and shoot tissues. To better visualize and confirm the distinct regulatory effects of the different S and Fe availabilities specifically on this key group of compounds, two additional heatmaps were generated, focusing solely on amino acids (Fig. 4c,d). In roots, a distinct accumulation pattern was observed and two clusters were identified. Cluster A displayed low amino acid levels and included all conditions except DS20 and DS50, which were grouped in Cluster B, characterized by high amino acid accumulation. In shoots, a similar pattern was observed, with the only difference being that the DS80 condition was also grouped in Cluster B (Fig. 4c,d). Among amino acids, asparagine emerged as a key metabolite due to its strong and consistent response to nutrient availability (Figs 4a,b, 5a,b). Specifically, asparagine concentrations show a clear and consistent decrease from 20 to 80 μM Fe under DS conditions, with the highest accumulation observed at DS20 in both roots and shoots (Fig. 5a,b).

**Fig. 6:**
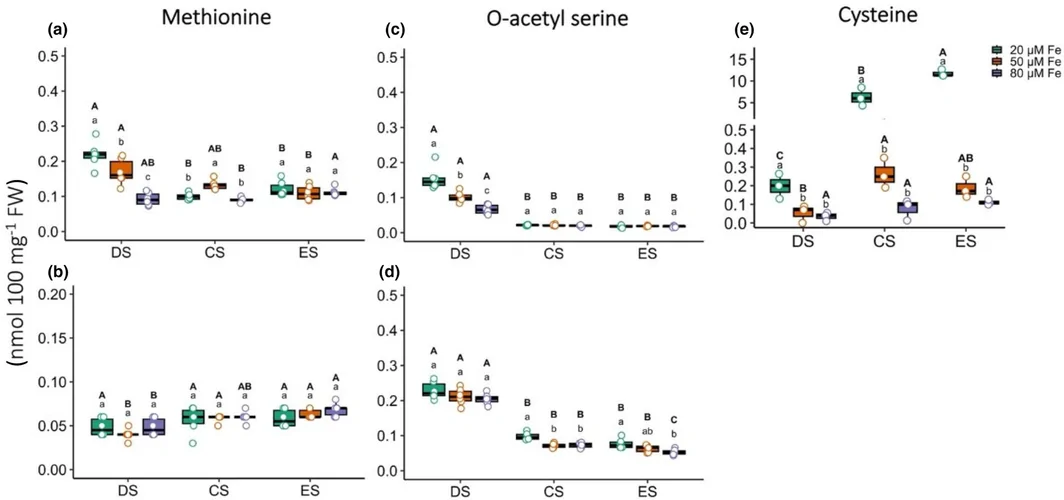
Changes in methionine, O‐acetyl serine (OAS), and cysteine concentrations in durum wheat in response to varying S and Fe supply. Plants were grown hydroponically for 2 wk at 0 (DS: deficient S supply), 1.2 (CS: control S supply), and 2.4 mM (ES: supra‐optimal S supply) sulfate, combined with 20, 50, and 80 μM Fe(III)‐EDTA. Root and shoot methionine (a, b), OAS (c, d), and root cysteine (e). Boxplots show the median, interquartile range, and data dispersion, while points represent biological replicates (*n* = 6). Data were analyzed using one‐way ANOVA followed by Tukey's *post hoc* test (*P* < 0.05). Different capital letters indicate significant differences among sulfate levels (S effect) within the same Fe concentration, while lowercase letters indicate significant differences among Fe(III)‐EDTA concentrations (Fe effect) within the same sulfate level (Tukey's HSD test, *P* < 0.05).

To further dissect S metabolism, the accumulation of methionine (Met) (Fig. 6a,b), *O*‐acetylserine (OAS) (Fig. 6c,d) and cysteine (Cys) (Fig. 6e) was analyzed. In roots, Met concentrations were significantly influenced by both Fe and S availability, but more markedly by Fe. The highest concentration was observed under combined deficiency of both nutrients (DS20); as Fe availability increased, concentrations dropped to 0.22, 0.17 and 0.09 nmol per 100 mg^−1^ FW (the lowest value), which consistently occurred whenever Fe was supplied at 80 μM. At this Fe concentration, Met levels decreased but were unaffected by S availability (Fig. 6a). This pattern mirrors the behavior of OAS under DS conditions (Fig. 6c), with Fe availability acting as the main driver; however, when S was supplied, OAS concentration dropped drastically regardless of Fe availability.

In shoots, Met accumulation was mainly driven by S availability, and the effect was significant under ES50 and ES80 conditions (Fig. 6c). On the other hand, for OAS concentration, the effect of S was also evident at the shoot level, although the influence of Fe became apparent only when S was available at concentrations above the optimal range (Fig. 6d).

Focusing on another important S metabolite, Cys accumulation showed a strong dependence on both nutrients. In roots, under CS and ES conditions, Cys reached its highest levels at low Fe supply, but was markedly repressed when Fe availability increased to 50 or 80 μM, reaching concentrations comparable to those observed under S deficiency. Under DS conditions, Cys levels remained consistently low, indicating that S availability is a prerequisite for Cys biosynthesis, while Fe sufficiency exerts a repressive effect (Fig. 6e).

**Fig. 7:**
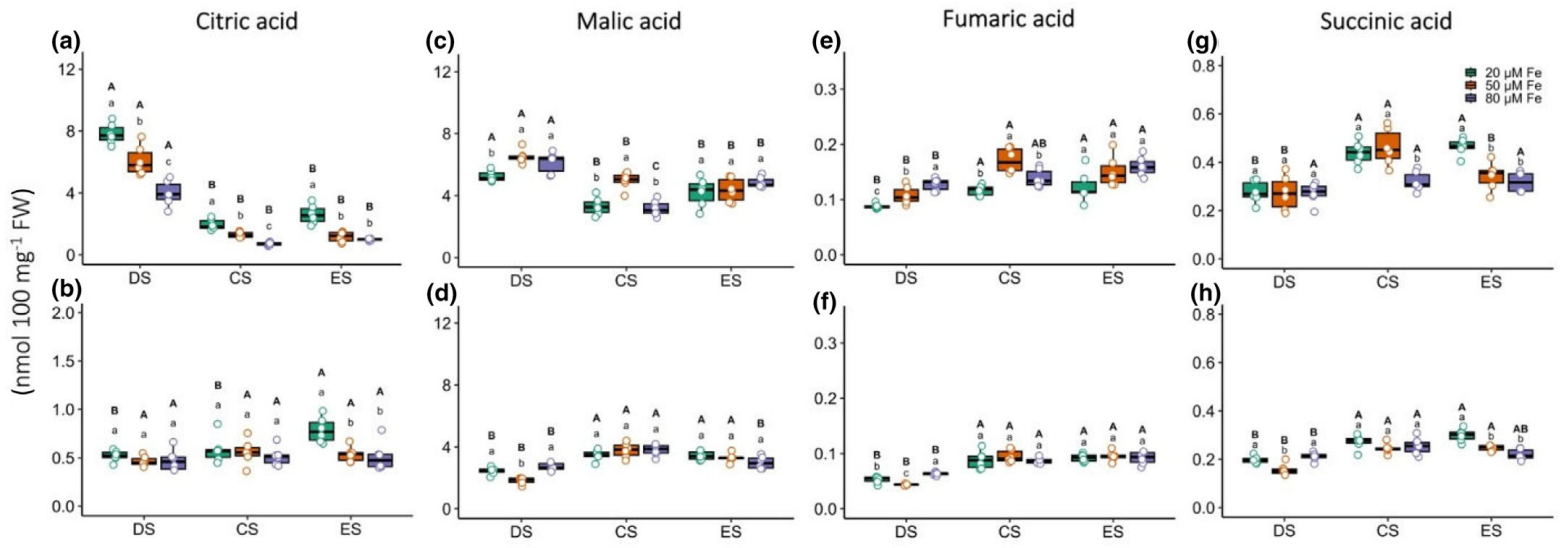
Changes in organic acid concentrations in durum wheat in response to varying S and Fe supply. Plants were grown hydroponically for 2 wk at 0 (DS: deficient S supply), 1.2 (CS: control S supply), and 2.4 mM (ES: supra‐optimal S supply) sulfate, combined with 20, 50, and 80 μM Fe(III)‐EDTA. Root and shoot concentrations of citric acid (a, b), malic acid (c and d), fumaric acid (e, f), succinic acid (g and h). Boxplots show the median, interquartile range, and data dispersion, while points represent biological replicates (*n* = 6). Data were analyzed using one‐way ANOVA followed by Tukey's *post hoc* test (*P* < 0.05). Different capital letters indicate significant differences among sulfate levels (S effect) within the same Fe concentration, while lowercase letters indicate significant differences among Fe(III)‐EDTA concentrations (Fe effect) within the same sulfate level (Tukey's HSD test, *P* < 0.05).

Organic acid metabolism was profoundly affected by the interaction between S and Fe, with citrate emerging as a central metabolic node (Fig. 7a). In roots, citric acid (CA) levels responded strongly to both S and Fe supply, showing a significant interaction between the two nutrients (Fig. 7a). Under DS conditions, CA content was highest and decreased progressively with increasing Fe availability. All Fe levels under DS formed distinct statistical groups, suggesting that Fe supply under S deficiency reduced CA accumulation. By contrast, under CS and ES conditions, CA content in roots remained significantly lower (2–3 nmol 100 mg^−1^ FW) and varied little among Fe levels. These treatments formed distinct groups from DS in the ANOVA test (uppercase letters), confirming that sufficient S availability suppressed CA accumulation in roots (Fig. 7a). In shoots, CA showed the same trend as Cys in roots: its concentration increased under Fe deficiency when S was at control levels, while remaining low under S deficiency. When Fe was raised to 50 or 80, CA accumulation was repressed, reaching levels typical of S starvation (Fig. 7b). Malic acid concentration varied significantly among treatments (Fig. 6c,d). In roots, plants grown under S deficiency (DS) showed higher values compared to CS and ES conditions, particularly at 20 and 50 μM Fe (Fig. 7c). In shoots, malate levels increased under CS, though differences among treatments were less marked (Fig. 7d). Fumaric acid concentration increased in roots under S deficiency (DS) with increasing Fe supply, whereas this trend was not observed in shoot tissues (Fig. 7e,f). By contrast, S deficiency significantly reduced succinic acid concentration in roots regardless of Fe availability, while under excess S (ES), the concentration of this organic acid decreased with increasing Fe levels in both roots and shoots (Fig. 7g,h).

**Fig. 8:**
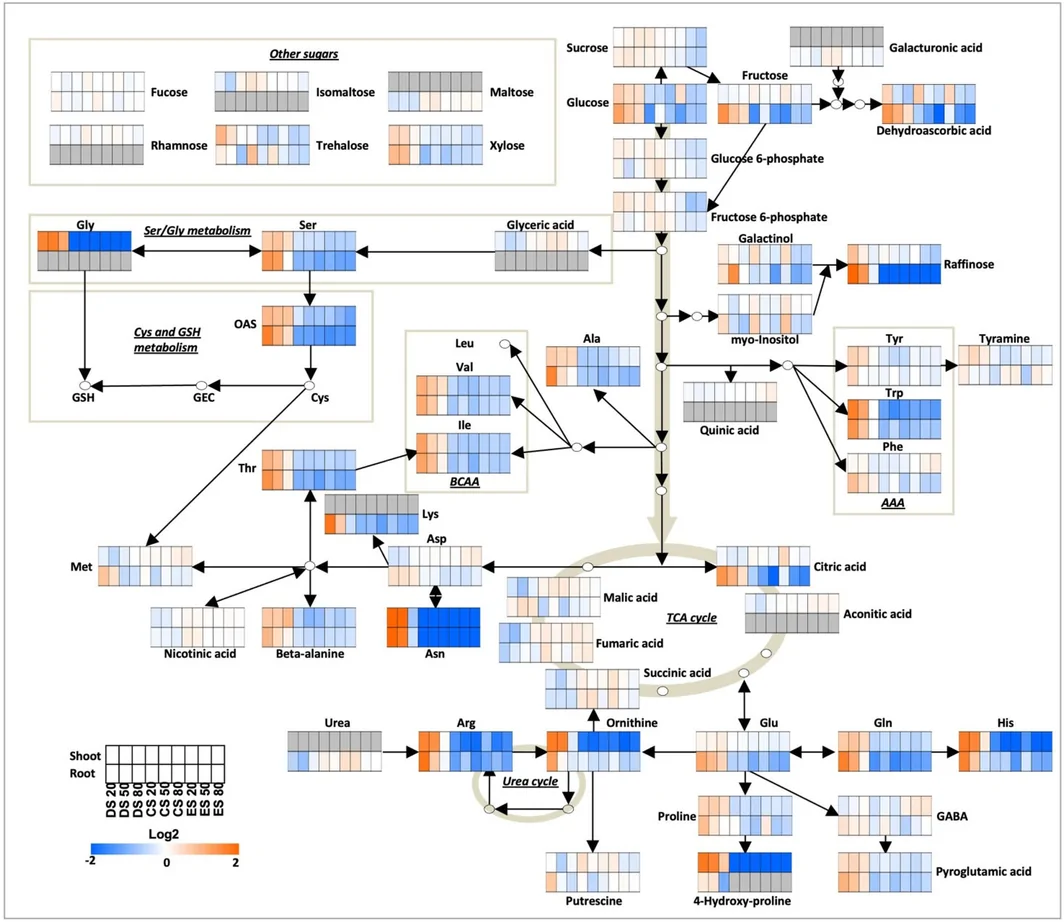
Synoptic overview of metabolic pathway changes in durum wheat under varying S and Fe supply. Plants were grown hydroponically for 2 wk at 0 (DS: deficient S supply), 1.2 (CS: control S supply), and 2.4 mM (ES: supra‐optimal S supply) sulfate, combined with 20, 50, and 80 μM Fe(III)‐EDTA. The heatmaps associated with each metabolite show relative abundance in shoots (upper row) and roots (lower row) across the nine experimental conditions. Log_2_ fold changes relative to the average across all conditions are shown in shades of red (high) and blue (low) according to the scale bar, while gray indicates metabolites that were not detected (below the detection limit). Data represent the mean values of six biological replicates (*n* = 6) per condition. AAA, aromatic amino acids; BCAA, branched‐chain amino acids; GABA, γ‐aminobutyric acid; TCA, tricarboxylic acid cycle.

An integrated overview of metabolite changes revealed that S deficiency promoted the accumulation of amino acids, polyamines and related compounds, in both organs, with a stronger effect in roots, while concurrently repressing sugars and organic acids. Increasing Fe supply progressively mitigated this metabolic signature, providing strong evidence for metabolic crosstalk between Fe and S nutrition (Fig. 8).

While most metabolites showed the same direction of change in both shoots and roots, the magnitude of change was generally more pronounced in roots. Conversely, certain metabolites were detected exclusively or exhibited stronger responses in shoots. Notably, several compounds, including fructose, tyramine, phenylalanine, Met, aspartate, malic acid, CA, and GABA, displayed divergent responses between shoots and roots, indicating that metabolic responses to nutrient stress are both tissue‐ and metabolite‐specific (Fig. 8).

**Fig. 9:**
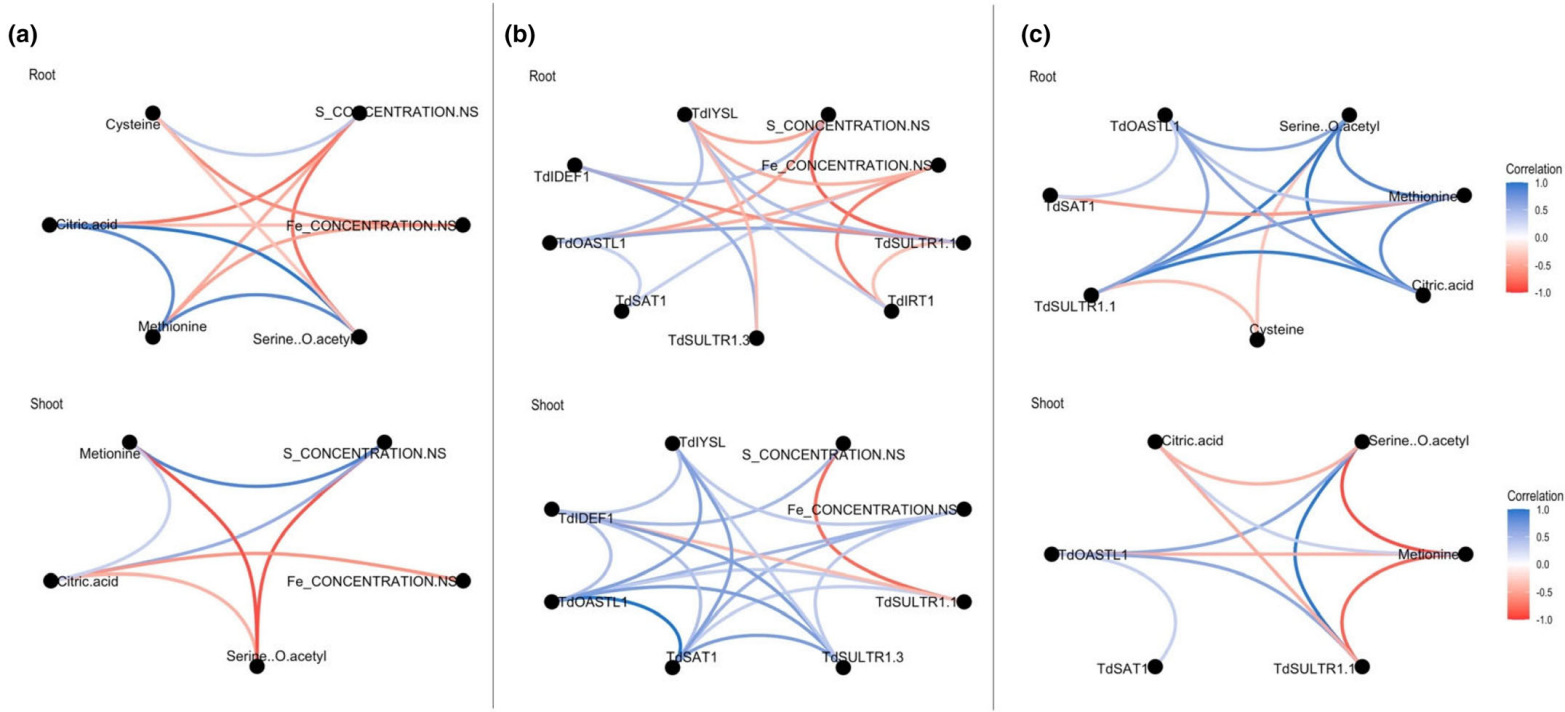
Tissue‐specific correlation networks of S‐ and Fe‐related variables. Panels show networks for roots (top) and shoots (bottom), highlighting positive (blue) and negative (red) interactions. Nodes represent individual biological variables, while edges indicate significant correlations and thickness reflects the correlation strength. Relationships between S and Fe supply and associated metabolites (a). Coordination among S and Fe‐responsive gene transcripts (b). Integrated network highlighting associations between gene expression and metabolite profiles (c).

Fig. 9 summarizes the correlation‐based relationships among S and Fe supply, metabolites, and transcriptional markers across the different Fe × S treatments, identifying the main components associated with the integrated response to S and Fe availability.

### Analysis of effect sizes for S, Fe, and S × Fe interaction across the strongest phenotypic, ionomic, transcriptional, and metabolic traits in durum wheat

**Fig. 10:**
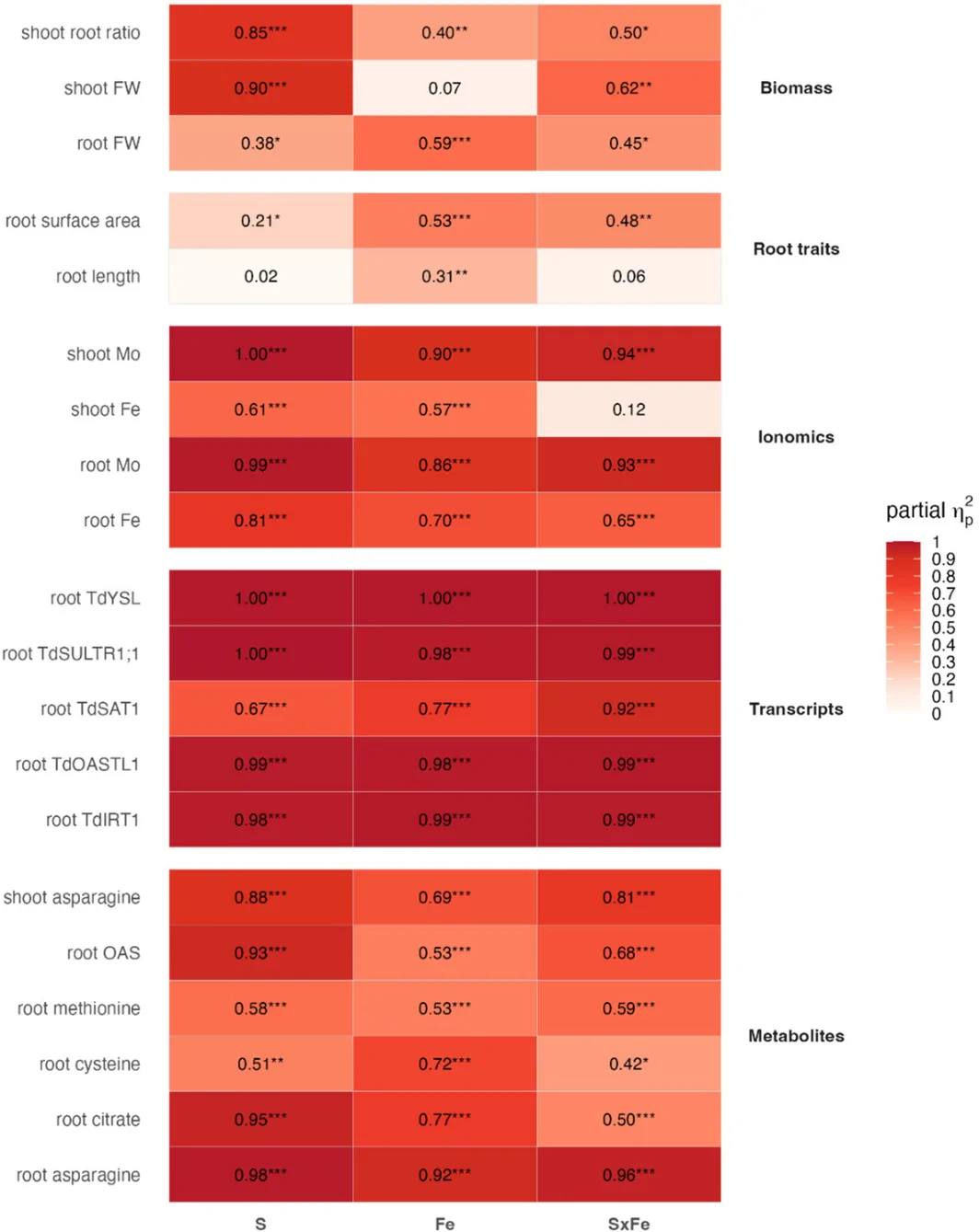
Summary heatmap of effect sizes for S, Fe, and S × Fe interaction across phenotypic, ionomic, transcriptional, and metabolic traits in durum wheat. Effect sizes were calculated as η_p_^2^ from two‐way ANOVA models including S supply (S), Fe availability (Fe), and their interaction (S × Fe) for each variable. Rows represent individual traits grouped into five categories: biomass, root traits, ionomics, transcripts, and metabolites. Columns indicate the effect size associated with S, Fe, and S × Fe, respectively. Cell colors represent the magnitude of the effect size, with darker colors indicating stronger effects. Values displayed within cells correspond to η_p_^2^; asterisks indicate statistical significance of the corresponding ANOVA term (*, *P* < 0.05; **, *P* < 0.01; ***, *P* < 0.001). Plants were grown hydroponically for 2 wk at 0 (DS: deficient S supply), 1.2 (CS: control S supply), and 2.4 mM (ES: supra‐optimal S supply) sulfate, combined with 20, 50, and 80 μM Fe(III)‐EDTA. This analysis summarizes the relative contribution of S, Fe, and their interaction to the variance observed across the measured traits.

To better synthesize the factorial structure of the dataset, we generated a summary heatmap of effect sizes derived from two‐way ANOVA across the most informative phenotypic, ionomic, transcriptional and metabolic traits (Fig. 10). This analysis revealed a clear stratification of S, Fe, and S × Fe effects across biological layers. Among growth traits, shoot fresh weight and the shoot : root ratio were primarily driven by S availability (0.90*** and 0.85***, respectively), whereas root fresh weight and root structural traits were more strongly influenced by Fe, particularly root surface area and root length. Regarding ionomic data, root Fe and both root and shoot Mo emerged as the most strongly integrated variables, showing large effect sizes for S, Fe and their interaction, while shoot Fe was influenced mainly by S and Fe (0.61*** and 0.57***, respectively). The strongest overall effects were observed for transcript abundance, with root TdYSL, TdSULTR1;1, TdOASTL1, and TdIRT1 all showing very large S, Fe and S × Fe contributions, indicating that the transcriptional response to Fe availability is tightly conditioned by S status and vice versa. Root TdSAT1 showed a stronger Fe and S × Fe effect (0.77*** and 0.92***, respectively) than S (0.67***). In the metabolite block, root and shoot asparagine, root OAS, and root citrate displayed the largest effect sizes, whereas both root methionine and cysteine showed more moderate but still significant responses.

## Discussion

### Reciprocal influence of S and Fe nutrition on plant phenotype and nutrient status

The combined analysis of plant biomass allocation and root morphological traits highlights the close interdependence between S and Fe nutrition in regulating durum wheat growth (Fig. 1). Our results indicate that S availability critically modulates the plant growth response to Fe supply, shaping both biomass partitioning and root system development. Under S deficiency (DS), increasing Fe supply strongly stimulated root biomass accumulation particularly at higher Fe levels (Fig. 1c), while the shoot‐to‐root ratio declined markedly (Fig. 1e). This shift toward root biomass allocation suggests an adaptive strategy in which plants prioritize belowground development to enhance nutrient acquisition under metabolic constraints (Robinson, 1994), although this pattern only partly agrees with previous reports and the broader literature remains inconclusive (Nikiforova *et al*., 2003). When S availability was optimal (CS), plant growth was more balanced, with both shoot and root biomass benefiting from increasing Fe supply (Fig. 1a,c), underscoring the requirement for adequate S to fully sustain Fe‐dependent metabolic and growth processes (Zuchi *et al*., 2012). Further S enrichment (ES) provided no additional growth benefit (Fig. 1a,c), consistent with previous observations in wheat (Zuchi *et al*., 2012). Overall, S availability modulated the impact of Fe deficiency on biomass allocation: S limitation constrained shoot growth and promoted a root‐dominated phenotype, whereas sufficient or excess S stabilized biomass partitioning across organs. Beyond biomass, the overall root system architecture also strongly responded to the combined effects of varied S and Fe availability (Fig. 1f). Under S‐deficient or optimal conditions, root architecture showed distinct clustering by Fe levels. However, under supra‐optimal S supply (ES), the influence of Fe on root morphology was largely overridden, suggesting S to become the main driver of root morphology under S abundance. The beneficial effect of supra‐optimal S supply, as reported in our recent work (Bruschini *et al*., 2025), may arise from enhanced support of S‐dependent metabolic processes and/or modulation of hormone‐regulated root development pathways (Lucena *et al*., 2015).

The ionomic clustering analysis further supports this coordinated control (Fig. 1g,h). In shoots, the clustering was mainly S‐driven (Fig. 1g). S deficiency led to the selective accumulation of Mo, Zn, and Mn, indicative of redox imbalance (Hawkesford, 2010; Rydz *et al*., 2021), while optimal S supported balanced profiles and supra‐optimal S elevated Mg, Cu, K, Fe, and Mn at higher doses. In roots, the S‐deficient treatments clustered distinctively, characterized by low concentrations of most elements but increased Mo and Fe accumulation under high Fe supply (DS80) (Fig. 1h). The consistent elevation of molybdenum under S deficiency likely reflects compensatory adjustments involving sulfate‐related pathways and redox‐associated enzymes under combined S and Fe limitation (Baxter, 2008; Koprivova & Kopriva, 2016). By contrast CS20, CS50, ES20, ES50 conditions showed enrichment in Ca, K, Mg, Zn, and Cu, indicative of a well‐coordinated nutrient status under adequate S supply (Fig. 1h). Collectively, these results indicate a tight functional coordination between Fe and S in plant homeostasis as well as effects on other nutrient ions: Fe availability becomes critical under S‐limiting conditions, whereas sufficient or excess S stabilizes physiological and ionomic responses and partially mitigates the effects of Fe deficiency.

### Metabolic hubs integrating S and Fe nutrition under combined deficiency

Metabolomic analysis revealed a pronounced accumulation of specific amino acids, particularly under combined S and Fe deficiency (DS20), indicating that amino acid metabolism constitutes a central hub in the metabolic response to nutrient stress (Bielecka *et al*., 2015). In wheat, however, both single and combined S deficiency induced a broad accumulation of amino acids, indicating that S availability plays a dominant role in shaping amino acid profiles under nutrient stress, as previously reported in tomato (Zuchi *et al*., 2015). Among these, OAS and Met accumulated strongly in roots under combined S and Fe deficiency, declining progressively with increasing Fe supply (Fig. 6). This response highlights a tight metabolic coupling between Fe availability and S assimilation under dual nutrient limitation (Astolfi *et al*., 2021). OAS functions as a key regulator and precursor in S metabolism by initiating the biosynthesis of Cys (Hubberten *et al*., 2012), while Met, synthesized downstream from Cys, serves as the precursor to S‐adenosylMet (SAM). Consistently, Cys levels were markedly reduced under S starvation (Fig. 6e). However, Cys abundance was also strongly modulated by Fe availability: the lowest Fe concentration (20 μM) triggered a substantial accumulation of Cys, whereas increasing Fe concentration to 50 or 80 μM reduced its levels to those typical of sulfate starvation (Fig. 6e). These results point to a direct regulatory mechanism whereby high Fe availability negatively feeds back on S assimilation, possibly to rebalance cellular redox and metabolic status, but they are also consistent with enhanced Cys consumption for Fe–S cluster assembly or other Fe‐chelating compounds that limit the accumulation of toxic free Fe.

Under adequate S conditions (CS), Fe deficiency induced the expression of S assimilation genes, including *TdSAT1* in both roots and shoots and *TdOASTL1* in roots (Fig. 2e–g), and promoted the accumulation of OAS and Cys (Fig. 6c,e), consistent with an increased demand for S‐containing metabolites to sustain redox balance (Ciaffi *et al*., 2013; Astolfi *et al*., 2021). By contrast, under combined S and Fe limitation, this coordinated response was disrupted: despite sustained *TdOASTL1* expression, Cys levels declined, indicating that Fe deficiency enhances S assimilation only when sulfate is sufficiently available. Co‐expression patterns between TdSULTR1;1 and TdOASTL1 further support a tight integration between sulfate uptake and assimilation under S limitation (Fig. 2).

The metabolic link between OAS, Met, and their downstream product S‐adenosylmethionine (SAM) highlights the broader integration of S metabolism with key regulatory pathways (Hesse & Hoefgen, 2003; Bürstenbinder *et al*., 2007; Hubberten *et al*., 2012). In parallel, citrate showed a pronounced accumulation under combined S and Fe deficiency, particularly in roots (Fig. 7a). While citrate accumulation under Fe deficiency is well documented (López‐Millán *et al*., 2000; Vigani *et al*., 2018), our results indicate that in durum wheat this response is strongly modulated by S availability and is maximized under dual limitation. Compared with tomato, where S deficiency attenuates citrate accumulation, durum wheat displays an opposite trend, with S limitation enhancing Fe deficiency‐induced citrate accumulation (Vigani *et al*., 2018; Coppa *et al*., 2023).

Citrate is a key tricarboxylic acid (TCA) cycle intermediate, and a well‐known chelator involved in Fe mobilization and transport. Although durum wheat relies on Strategy II mechanisms for Fe uptake, citrate accumulation may contribute to Fe mobilization and internal redistribution when phytosiderophore biosynthesis is constrained by limited S availability, thus acting as an alternative or complementary mechanism to sustain Fe homeostasis. Notably, citrate accumulation closely paralleled the behavior of OAS and Met in roots (Figs 7, 6), and correlation network analysis revealed strong positive associations among these metabolites and negative correlations with both Fe and S availability (Fig. 9a). Besides integration of TdSULTR1;1 and TdOASTL1 genes into the root metabolic network revealed strong positive correlations among CA, Met, and OAS, together with TdSULTR1;1 and TdOASTL1 (Fig. 9b), supporting the existence of a coordinated metabolic–transcriptional hub linking S assimilation with Fe‐dependent metabolism. This convergence across metabolic and transcriptional layers highlights citrate as a central component of the coordinated response to Fe/S imbalance, reflecting a tight coupling between carbon metabolism, S assimilation, and Fe homeostasis. Rather than indicating a direct regulatory role, these findings point to citrate as a robust metabolic signature of Fe/S interaction, providing new insight into how central metabolism is reconfigured under combined nutrient stress.

Beyond its established role in Fe chelation and transport, citrate has also been proposed to participate in broader metabolic coordination processes under nutrient stress. Given the central role of mitochondria in both citrate production and Fe–S cluster biogenesis, citrate accumulation may further reflect mitochondrial perturbations triggering retrograde signaling under nutrient stress. Overall, our data primarily support a role for citrate as a key metabolic component associated with Fe/S crosstalk, linking central carbon metabolism with S assimilation and Fe homeostasis, while its potential regulatory or signaling functions remain to be clarified in future studies. Direct functional validation, including perturbation of citrate metabolism and analysis of TOR activity or Fe–S cluster‐related markers, will be required to establish whether citrate has any causal regulatory role beyond its metabolic and chelation‐related associations.

### Sulfur‐driven nitrogen reallocation and potential implications for food safety

Among all metabolites analyzed (Fig. 4a–d), free asparagine displayed the most pronounced and agronomically relevant response to nutrient availability (Fig. 5). Sulfur deficiency consistently triggered a strong accumulation of asparagine in both roots and shoots (Fig. 5), confirming previous observations in wheat and other cereals (Raffan *et al*., 2020). Importantly, this response was further amplified when S and Fe deficiencies occurred simultaneously, revealing a synergistic interaction between the two nutrient stresses on N metabolism. When S is inadequate, protein synthesis is constrained (due to limited Cys and Met availability), leading plants to redirect excess of N into free amino acids, particularly asparagine, which serves as a major N storage and transport compound (Oddy *et al*., 2020; Raffan *et al*., 2020; Aarabi *et al*., 2021).

Our data indicate that this S‐driven N reallocation is highly active in durum wheat and further intensified by concurrent Fe deficiency. This mirrors observations in tomato, where the combined S and Fe deficiency caused an increase in N‐rich amino acids like asparagine, glutamine, arginine, and lysine (Zuchi *et al*., 2015). By contrast, Fe deficiency alone, under adequate S supply, did not induce substantial asparagine accumulation in vegetative tissues (Fig. 5), consistent with reports in sugar beet leaves showing that Fe deficiency can alter amino acid profiles without necessarily increasing asparagine (López‐Millán *et al*., 2000). These findings highlight S deficiency as the primary driver of asparagine accumulation in vegetative tissues, with Fe deficiency acting as a powerful modulator of its magnitude rather than its direction. We therefore suggest that S deficiency imposes the main limitation on protein synthesis, while Fe deficiency reduces growth‐driven N demand, together causing an overload of free amino acids. Asparagine is the main precursor of acrylamide, a neurotoxic and potentially carcinogenic compound, through the Maillard reaction during high‐temperature processing conditions used in cereal‐based food production (Muttucumaru *et al*., 2008; Halford *et al*., 2012). It is still unclear to what extent asparagine is imported into the wheat grain, and how much such import contributes relative to *in situ* synthesis within grain tissues (Oddy *et al*., 2020). Nevertheless, substantial evidence indicates that S deficiency can markedly increase free asparagine accumulation in wheat grains (reviewed in Raffan *et al*., 2020). Accordingly, while our vegetative‐stage study does not allow direct conclusions on grain composition or acrylamide risk, it suggests the possibility that co‐occurring S and Fe deficiencies may represent a relevant risk factor influencing the acrylamide‐forming potential of durum wheat products. This hypothesis warrants further investigation under reproductive‐stage and agronomically relevant conditions.

Aside from asparagine, other amino acids in our study also showed notable changes (Fig. 4c,d). In particular, glutamine and arginine increased under S deficiency, indicating a preferential accumulation of N‐rich, non‐S amino acids when S is limiting, as N that cannot be incorporated into S‐containing amino acids (Cys, Met), is instead redirected into alternative N‐rich amino acids (Zuchi *et al*., 2015). By contrast, Fe deficiency is known to affect amino acid profiles in a different way, often increasing specific stress‐related amino acids (such as proline or alanine) while decreasing others (Zuchi *et al*., 2015). In this work, plants subjected to combined deficiencies predominantly displayed an S‐driven pattern, characterized by the buildup of N‐rich amino acids, which clearly dominated over an Fe‐driven signature. This indicates that S availability is a primary driver of free amino acid composition, with Fe deficiency modulating the magnitude but not the direction of these changes when both stresses co‐occur.

Our results support the coordinated regulation of S and Fe metabolism and indicate that combined stress conditions trigger a broad reallocation of nitrogen, likely as an adaptive strategy to sustain metabolism. Under S‐sufficient conditions, Fe deficiency activated S assimilation pathways, with increased expression of TdSAT1 and TdOASTL1 (Fig. 2e–g) and accumulation of OAS and Cys (Fig. 6c,e), suggesting enhanced flux toward S‐containing amino acids. By contrast, under combined S and Fe deficiency, this coordination was disrupted: despite transcriptional activation, limited sulfate availability constrained Cys synthesis, promoting a shift toward non‐S amino acids. This transcriptional–metabolic uncoupling likely underlies the accumulation of N‐rich compounds under S deficiency and highlights the central role of S availability in controlling N partitioning under combined nutrient stress.

### Tissue‐specific metabolic plasticity under S and Fe stress

Overall, the plant's metabolic response to S deficiency was extensive, highlighting the central role of S in maintaining whole‐plant metabolic balance (Figs 4, 8). Increasing Fe availability partially alleviated these effects, confirming a tight Fe/S crosstalk in regulating metabolic homeostasis. A key feature of this response was its pronounced tissue specificity, with both coordinated and organ‐specific metabolite changes, consistent with adaptive responses reported under environmental stress (Garriga *et al*., 2014; Forieri *et al*., 2016; Zenzen *et al*., 2024). The larger metabolic shifts often observed in roots likely reflect their role as the primary site of nutrient uptake, where local metabolic adjustments are triggered immediately by external nutrient fluctuations. Conversely, shoot‐specific accumulation of certain compounds highlights the unique metabolic priorities of photosynthetic tissues: sugars derived from primary production, such as rhamnose, or starch degradation such as isomaltose, as well as organic acids, linked to the TCA cycle, glycolysis and the shikimate pathway, including aconitic, glyceric, and quinic acids, and stress‐related amino acids (e.g. glycine, histidine, and ornithine) showed distinct patterns in shoots. The shoot‐specific accumulation of these metabolites likely arises from the high availability of carbon skeletons and the enhanced activation of stress‐related metabolic pathways in photosynthetic tissues. Opposite trends between organs for metabolites such as fructose, tyramine, phenylalanine, Met, aspartate, malic acid, CA, and GABA further emphasize that metabolic adjustments are both tissue‐ and metabolite‐specific, likely reflecting distinct physiological roles: shoots prioritize carbon metabolism and stress signaling, whereas roots are mainly engaged in nutrient acquisition and associated energy and amino acid metabolism, potentially combined with altered metabolite exchange between organs under nutrient limitation.

This pronounced tissue‐specific plasticity in response to Fe and S imbalance is also evident at the transcriptional level. In shoots, TdSULTR1;3, previously reported to be induced by Fe deficiency (Ciaffi *et al*., 2013), showed increased expression with rising S supply under Fe deficiency (Fig. 2d), likely supporting sulfate redistribution toward Fe‐dependent processes, while enhanced expression of TdSULTR1;1 under S deficiency (Fig. 2b) points to intensified sulfate translocation between organs, in line with previous reports (Abdallah *et al*., 2010; Shinmachi *et al*., 2010; Honsel *et al*., 2012; Ciaffi *et al*., 2013). In contrast to TdSULTR1;1 and TdOASTL1, TdSULTR1;3 reached its highest expression when S was sufficient and Fe abundant (Fig. 2) and showed no significant correlation with metabolic parameters in either organ (Fig. 9), suggesting that sulfate uptake and redistribution are modulated by distinct molecular modules depending on whether plants experience S or Fe deficiency.

Iron acquisition genes further emphasized tissue‐specific responses: tight co‐expression of TdIRT1 and TdYSL under low Fe supply characterized a high‐affinity Fe uptake system that was rapidly downregulated as Fe became abundant (Fig. 3), reflecting tight modulation of Fe homeostasis (Kobayashi & Nishizawa, 2012). Sulfur deficiency partially suppressed this response, whereas supra‐optimal S supply enhanced Fe uptake and translocation, together with increased TdYSL and TdIDEF1 expression in shoots (Fig. 3b,d). Notably, TdIDEF1 displayed non‐canonical regulation, peaking at high Fe concentrations (Fig. 3a,b), consistent with a broader integrative role beyond classical Fe deficiency sensing (Kobayashi *et al*., 2007; Coppa *et al*., 2025), and its positive association with TdSULTR1;3 in both roots and shoots (Fig. 9) further supports functional integration between S status and Fe acquisition pathways.

### A layered view of Fe/S crosstalk in durum wheat

The effect‐size heatmap condenses the central outcome of the factorial design: Fe/S crosstalk was expressed most strongly at the transcriptional and metabolic levels, whereas Fe exerted a comparatively larger influence on root growth‐related traits. Sulfur accounted for most of the variance across transcriptional and metabolic responses, suggesting that it largely sets the amplitude of the response, while Fe contributed more strongly to root developmental adjustment. At the same time, the consistently large S × Fe effects for root Fe, Fe‐ and S‐homeostasis transcripts, and selected metabolites indicate that many responses cannot be interpreted as the sum of independent S and Fe effects but instead reflect coordinated adjustment under combined nutrient constraints. This is consistent with previous work showing that S availability strongly conditions Fe acquisition, Fe accumulation, and the expression of S‐assimilation and Fe‐homeostasis genes in cereals, including durum wheat (Zuchi *et al*., 2012; Ciaffi *et al*., 2013; Zamboni *et al*., 2017; Astolfi *et al*., 2021). Within the metabolic layer, citrate and OAS emerged as the strongest signatures of Fe/S integration, together with methionine and asparagine also showing robust responses; by contrast, cysteine was comparatively less informative at the effect‐size level. This pattern is biologically plausible because OAS is a central sulfur‐responsive metabolite and precursor of cysteine biosynthesis (Hubberten *et al*., 2012), citrate has repeatedly been associated with Fe deficiency and Fe‐ and S‐dependent metabolic adjustment (Vigani *et al*., 2018), and S deficiency is well‐known to promote asparagine accumulation and N reallocation in wheat and other species (Zuchi *et al*., 2015; Raffan *et al*., 2020). A major conceptual outcome of the factorial design is that S and Fe do not contribute symmetrically to the observed responses. Across multiple biological layers, S availability emerged as the primary determinant of the metabolic and transcriptional state, whereas Fe availability mainly modulated the magnitude of these responses. This pattern was evident not only from the effect‐size analysis but also from the coordinated trajectories observed across the Fe gradient under constant S deficiency. In particular, OAS, methionine, citrate and asparagine reached their highest levels under combined Fe and S limitation and progressively declined as Fe availability increased, indicating that Fe availability attenuates rather than overrides the S deficiency response program. The factorial matrix further revealed that many of the strongest responses could not be explained by either nutrient alone. Instead, several transcripts and metabolites displayed large S × Fe effects, demonstrating that the combined nutritional context generates emergent properties that are not predictable from single‐deficiency experiments. The coordinated behavior of OAS, methionine, citrate and asparagine, together with their integration into transcriptional networks involving sulfate assimilation and Fe‐homeostasis genes, supports the existence of a non‐additive Fe/S‐responsive module operating primarily in roots. Importantly, the value of the present study does not lie in identifying a single regulatory mechanism, but in resolving how Fe and S jointly shape biological responses across molecular and physiological scales. By separating nutrient‐specific effects from interaction effects, the factorial framework provides a mechanistic level of interpretation that is not accessible through conventional deficiency‐vs‐control comparisons. In this respect, the identified Fe/S‐responsive module represents a testable framework for future functional studies aimed at dissecting the causal links between S assimilation, Fe homeostasis and central metabolism.

### Conclusions

Our factorial analysis reveals extensive reciprocal interactions between Fe and S nutrition in durum wheat and identifies a robust set of phenotypic, ionomic, transcriptional, and metabolic signatures associated with their combined availability. Sulfur supply emerged as the dominant modulator of the magnitude and direction of Fe‐responsive traits, while citrate, OAS, methionine, cysteine, and asparagine consistently marked conditions of strong Fe/S integration. Because these inferences are based on association patterns across multiple biological layers, they should be interpreted as hypothesis‐generating rather than causal. The present study therefore provides a systems‐level framework and a prioritized set of candidate processes for future functional work on Fe/S crosstalk in cereals.

## Competing interests

None declared.

## Author contributions

SA conceived and designed the study. EC, MW, MM, GQ and AB performed the research. EC, MW and GV analyzed the data. SA, EC and GV wrote the original draft; SA, EC, RH, YP, SC and GV revised and edited the manuscript. All authors approved the manuscript.

## Disclaimer

The New Phytologist Foundation remains neutral with regard to jurisdictional claims in maps and in any institutional affiliations.

## Supporting information


**Fig. S1** Morphological parameters of the root system of durum wheat plants.
**Fig. S2** Concentrations of K, Mg, Ca, Zn, Fe, Cu, Mn, and Mo in root and shoot tissues of durum wheat.
**Table S1** Primer pairs used for the isolation of the full‐length cDNA of durum wheat genes.
**Table S2** Gene sequence details.
Please note: Wiley is not responsible for the content or functionality of any Supporting Information supplied by the authors. Any queries (other than missing material) should be directed to the *New Phytologist* Central Office.

## Data Availability

The data that supports the findings of this study are available in Tables S1, S2, Figs S1 and S2.
