# Supplementary material for: A factorial multi‐layer analysis reveals non‐additive sulfur–iron interactions shaping metabolic and transcriptional responses in durum wheat
Source: New Phytol. 2026 Aug 13;252(1):192–208. doi: 10.1111/nph.71507 (PMC13539956; doi:10.1111/nph.71507)
Supplement: Supplementary file 1 [file NPH-252-192-s001.pdf]

New Phytologist Supporting Information

Article title: **A factorial multi-layer analysis reveals non-additive sulfur–iron interactions shaping metabolic and transcriptional responses in durum wheat**

Authors: Eleonora Coppa[1], Mutsumi Watanabe[2,3], Moez Maghrebi[4], Giulia Quagliata[1], Alessandro Bruschini[1], Rainer Hoefgen[3], Youry Pii[5], Stefano Cesco[5], Gianpiero Vigani[4], Stefania Astolfi[1*]

Article acceptance date: 7 July 2026

Table S1: Primer pairs used for the isolation of the full-length cDNA of durum wheat genes

Target gene	Primers	Oligo sequence (5' to 3')	Synthesis scale (use drop-down)	Purification (use drop-down)
TdSULTR1.1	TdSULTR1.1.For1	CTATGAGACAGCCTACCTTATATG	50N	DSL
	TdSULTR1.1.Rev1	CAACCGCAATGAGCAAGC	50N	DSL
TdSULTR1.3	TdSULTR1.3.For1	ATGTCAGTGGTGGTTCTTC	50N	DSL
	TdSULTR1.3.Rev1	ATTGCTGCTTCGTAGTCC	50N	DSL
TdSAT1	TdSAT1.For1	GTCTCCATCCTCCACCAC	50N	DSL
	TdSAT1.Rev1	GCACATCTATCAGCACCAC	50N	DSL
TdOASTL1	TdOASTL1.For1	GCCCAGAGACCCGAGAAC	50N	DSL
	TdOASTL1.Rev1	AACAGCACCGACGACAGG	50N	DSL
TdIRT1	TdIRT1.For1	TATGCATTGCCCCCTAGAAA	50N	DSL
	TdIRT1.Rev1	ATGAACGGGAAAACCATCAC	50N	DSL
TdIDEF1	TdIDEF1.For1	TGCGGTTCTGTAAATATTCTCG	50N	DSL
	TdIDEF1.Rev1	CATCATAGCACGTCCAAAGTCC	50N	DSL
TdYSL	TdYSL.For1	CGCAAGCAGGTTGACAACAAAGC	50N	DSL
	TdYSL.Rev1	TGGCGTTGAAACTCCTCAAGCG	50N	DSL

Table S2: Gene Sequence Details. Putative durum wheat homologs were identified through BLAST searches against the Triticum aestivum reference genome, using thresholds of >90% query coverage, >85% sequence identity, and an E-value < 1×10^{-100}. Gene assignments were supported by phylogenetic evidence and the high sequence similarity (>95%) between durum wheat and bread wheat genomes. Ensembl transcript identifiers and genomic locations of the selected sequences are provided.

Gene	GeneBank ID (NCBI)	Transcript ID (Ensembl)	Genome Position (Ensembl)	Key Reference & Homolog
TdSULTR1.1	XM_044520384	TraesCS4D03G0628400	4D:434643286-434649517	Ciaffi et al., 2013; *TaSULTR1.1*
TdSULTR1.3	XM_044525606	TraesCS5A03G0585300	5A:445364770-445371324	Ciaffi et al., 2013; *TaSULTR1.3*
TdSAT1	XM_044561005	TraesJAG1B03G00360890	1B:609076725-609077935	Ciaffi et al., 2013; *TaSAT1*
TdOASTL1	XM_044499481	TraesCS3D03G0172300	3D:44118677-44122529	Ciaffi et al., 2013; *TaOASTL1*
TdIDEF1	XM_044566988	TraesCS7B02G230100	7A:481247738-481251192	Saidi et al., 2025; *TaIDEF1*
TdIRT1	XP_044361150	TraesCS4A02G403300	4A:676813027-676817607	Saidi et al., 2025; *TaIRT1*
TdYSL1	XM_044557611	TraesCS6B02G283800	6B:389932401-389939782	Saidi et al., 2025; *TaYSL1*

References

Ciaffi M, Paolacci AR, Celletti S, Catarcione G, Kopriva S, Astolfi S. 2013. Transcriptional and physiological changes in the S assimilation pathway due to single or combined S and Fe deprivation in durum wheat (*Triticum durum* L.) seedlings. *Journal of Experimental Botany* **64:** 1663–1675.

Saidi MN, Rebai O, Hachani F, Vigani G, Astolfi S. Transcriptomics Data Mining to Identify Novel Regulatory Genes of Iron Uptake in Drought-Stressed Wheat. *International Journal of Molecular Sciences*. 2025; **26(22):**10955. https://doi.org/10.3390/ijms262210955

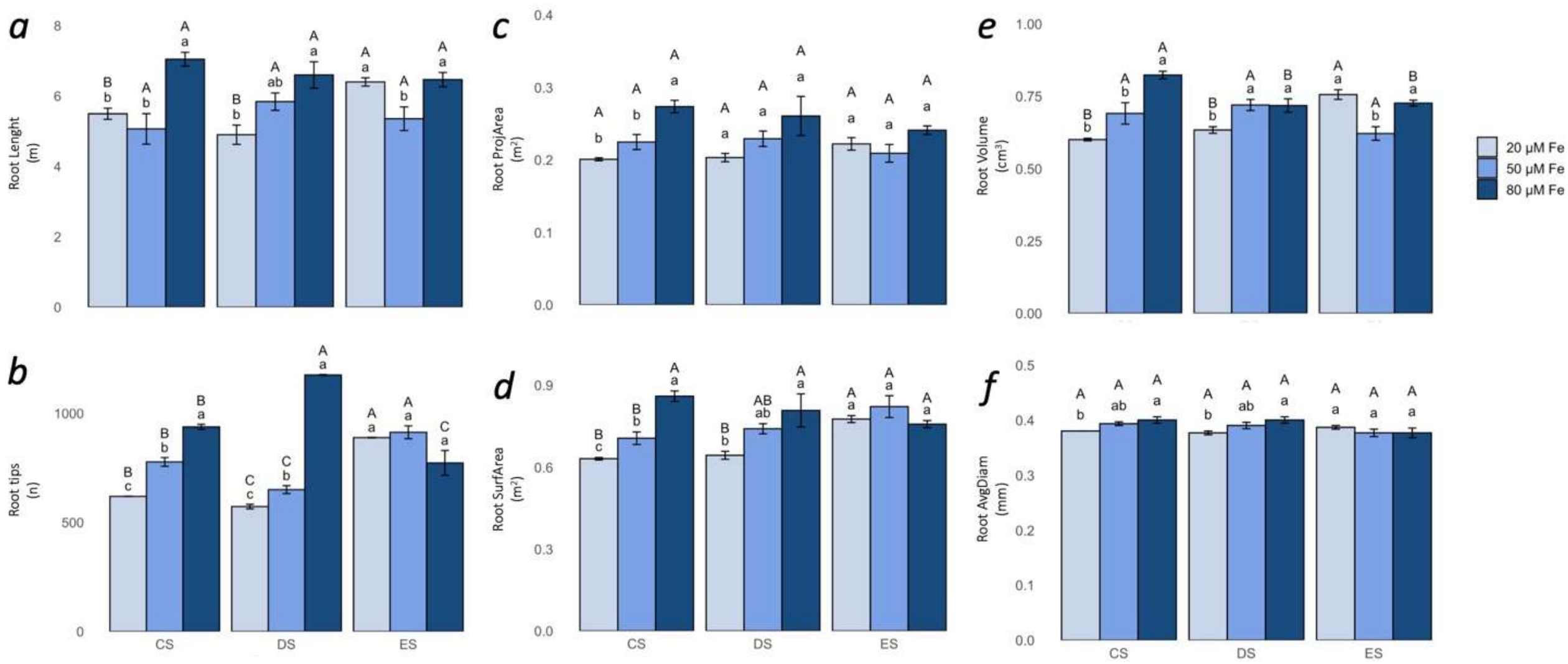

Supplementary Figure 1. Morphological parameters of the root system of durum wheat plants: length (a), number of tips (b), projected area (c), surface area (d), volume (e), average diameter (f). Plants were grown hydroponically for two weeks at 0 (DS: deficient S supply), 1.2 (CS: control S supply), and 2.4 mM (ES: supra-optimal S supply) sulfate, combined with 20, 50, and 80 µM Fe(III)-EDTA. Data are means ± SD (n = 3 biological replicates). Statistical significance ($p < 0.05$) was determined via one-way ANOVA and Tukey's test. Capital letters denote differences among S levels at each Fe dose; lowercase letters denote differences among Fe doses at each S level.

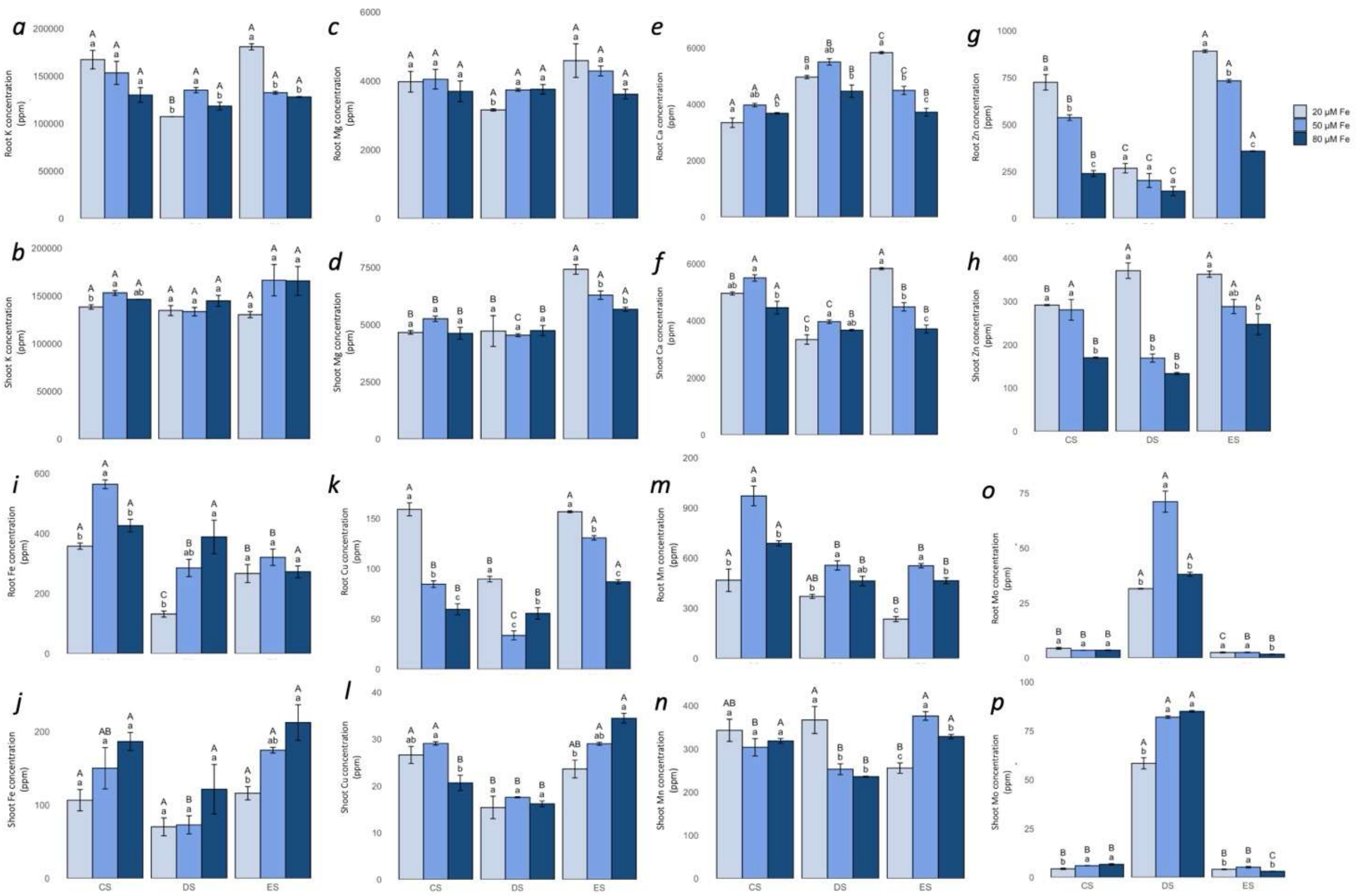

Supplementary Figure 2. Concentrations of K (a, b), Mg (c, d), Ca (e, f), Zn (g, h), Fe (i, j), Cu (k, l), Mn (m, n), and Mo (o, p) in root (a, c, e, g, i, k, m, o) and shoot (b, d, f, h, j, l, n, p) tissues of durum wheat. Plants were grown hydroponically for two weeks at 0 (DS: deficient S supply), 1.2 (CS: control S supply), and 2.4 mM (ES: supra-optimal S supply) sulfate, combined with 20, 50, and 80 µM Fe(III)-EDTA. Data are means ± SD (n = 3 biological replicates). Statistical significance ($p < 0.05$) was determined via one-way ANOVA and Tukey’s test. Capital letters denote differences among S levels at each Fe dose; lowercase letters denote differences among Fe doses at each S level.
